# Supplementary material for: The UK Soft Drinks Industry Levy and childhood hospital admissions for asthma in England
Source: Nat Commun. 2024 Jun 10;15:4934. doi: 10.1038/s41467-024-49120-4 (PMC11164966; doi:10.1038/s41467-024-49120-4)
Supplement: Supplementary file 1 — Supplementary Software 1 [file 41467_2024_49120_MOESM1_ESM.pdf]

The UK Soft Drinks Industry Levy and childhood hospital admissions for asthma in England

Supplementary Software

Nina T Rogers, Steven Cummins, Catrin P Jones, Oliver T Mytton, Chrissy h Roberts, Seif O. Shaheen, Syed Ahmar Shah, Aziz Sheikh, Martin White, Jean Adams

Summary

This supplement provides copies of all R code and associated outputs that were created in the study. The original analyses used a combination of R Markdown (for analyses) and R scripts (Providing functions and data preparation). The data used in this study cannot be shared by the authors, but is available from NHS Digital Data Access Request Service.

Contents

- Time Series Analyses — 02
-
- By Age — 02
  - 05 - 18 Years — 02
  - 05 - 09 Years — 14
  - 10 - 14 Years — 26
  - 15 - 18 Years — 40
-
- By Deprivation Quintile — 54
  - IMD-5 - Least Deprived — 54
  - IMD-4 - Less Deprived — 66
  - IMD-3 - Mid Deprived — 78
  - IMD-2 - More Deprived — 90
  - IMD-1 - Most Deprived — 102
-
- R Code Used to Prepare Data — 114
- R Functions for Age Analysis — 118
- R Functions for Deprivation Analysis — 122

Asthma Analysis (05-18 years)

R code

- Code : Nina Rogers (Nina.Rogers@mrc-epid.cam.ac.uk)
- Code review : Chrissy h. Roberts (chrissy.roberts@LSHTM.ac.uk)

Data Prep and Functions Load

```
source("001_asthma_data_prep_age_sep.R")
source("002_asthma_age_functions.R"
```

Chart admissions data

This provides a simple eyeball test of the data trend across time.

```
ggplot(age05_18, aes(x=measurement_date, y=admission_prop )) +
geom_smooth() +
xlab("")+
  ylab("Admissions per 100,000 population, per month")
```

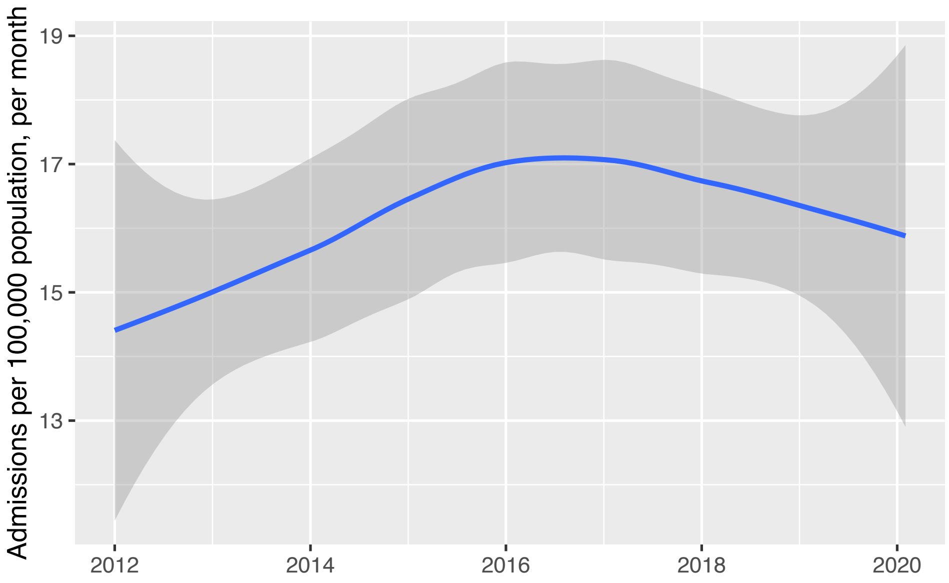

Create the time and intervention variables

This includes definition of time_months (study time), announcement.trend (change in trajectory post announcement) and imp.trend (change in trajectort post implementation).

```
age05_18$time_months <-c(1:98)
age05_18$announcement.trend <- c(rep(0,51),1:47)
age05_18$imp.trend <- c(rep(0,75),1:23)
age05_18 <- age05_18 %>% select(measurement_date, time_months, everything())
```

Run OLS analysis

```
age05_18.ols<-model_ols(df = age05_18)
summary(age05_18.ols)
```

```
Call:
lm(formula = admission_prop ~ time_months + announcement.trend +
    September + October + November + April + August + imp.trend,
    data = df)

Residuals:
    Min      1Q  Median      3Q     Max
-3.8016 -1.2052 -0.1498  0.9010  8.0770

Coefficients:
                   Estimate Std. Error t value Pr(>|t|)
(Intercept)        14.13468    0.54554  25.910  < 2e-16 ***
time_months         0.05201    0.01601   3.248  0.00164 **
announcement.trend -0.07266    0.04342  -1.673  0.09779 .
September           7.39889    0.72851  10.156  < 2e-16 ***
October             3.48432    0.72876   4.781 6.85e-06 ***
November            3.73759    0.72913   5.126 1.71e-06 ***
April              -3.35603    0.72915  -4.603 1.38e-05 ***
August             -7.07268    0.72839  -9.710 1.27e-15 ***
imp.trend          -0.02451    0.06968  -0.352  0.72579
---
Signif. codes:  0 '***' 0.001 '**' 0.01 '*' 0.05 '.' 0.1 ' ' 1

Residual standard error: 1.931 on 89 degrees of freedom
Multiple R-squared:  0.781, Adjusted R-squared:  0.7614
F-statistic: 39.69 on 8 and 89 DF,  p-value: < 2.2e-16
```

Run Durbin-Watson Test

```
age05_18.dwt<-model_dwt(age05_18.ols)
age05_18.dwt
```

```
 lag Autocorrelation D-W Statistic p-value
   1     0.149017833      1.696173   0.084
   2    -0.019948796      2.027395   0.980
   3     0.024029998      1.935229   0.692
   4    -0.004811642      1.986616   0.980
   5    -0.119161520      2.214031   0.210
   6    -0.182408222      2.332389   0.042
   7    -0.064013865      1.899009   0.926
   8    -0.098939528      1.941353   0.818
   9    -0.077199669      1.887759   0.910
  10    -0.089098891      1.905980   0.812
  11    -0.034742575      1.770369   0.806
  12     0.034258007      1.600848   0.152
  13     0.060886923      1.535658   0.252
  14     0.046069066      1.554933   0.380
  15     0.009084128      1.618691   0.616
  16    -0.012304352      1.658075   0.878
  17    -0.045418363      1.724207   0.860
  18    -0.167131408      1.960427   0.156
  19    -0.064963966      1.738552   0.650
  20     0.036639667      1.491158   0.598
 Alternative hypothesis: rho[lag] != 0
```

Chart acf of ols

```
graph.ols.acf.plots(model = age05_18.ols)
```

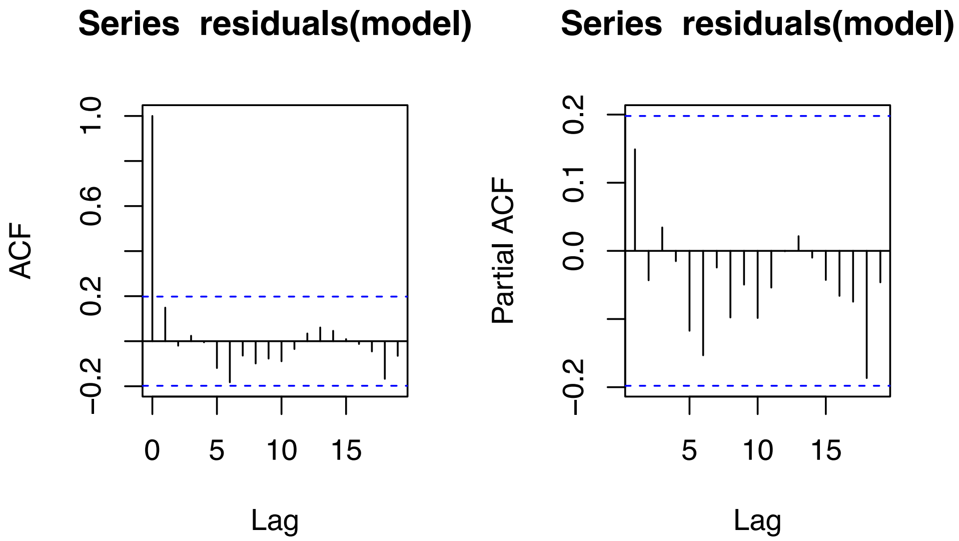

Run gls models with a variety of values of p and q

The DWT and ACF chart suggestlag of 1 may be optimal.

Run a variety of values here to provide belt and braces. Using values 1,2,3,4,6,8 and 12, allows for various cyclical calendar-relevant lags. fit.01 provides a linear relationship between x and y with no lag, whilst fit.02 provides a time-squared function without lag.

```
fit.01<-gls.modeller(df = age05_18,timesquared = FALSE,timecubed = FALSE,p=NULL,q=NULL)
fit.02<-gls.modeller(df = age05_18,timesquared = TRUE,timecubed = FALSE,p=NULL,q=NULL)

fit.03<-gls.modeller(df = age05_18,timesquared = FALSE,timecubed = FALSE,p=1,q=NULL)
fit.04<-gls.modeller(df = age05_18,timesquared = FALSE,timecubed = FALSE,p=2,q=NULL)
fit.05<-gls.modeller(df = age05_18,timesquared = FALSE,timecubed = FALSE,p=3,q=NULL)
fit.06<-gls.modeller(df = age05_18,timesquared = FALSE,timecubed = FALSE,p=4,q=NULL)
fit.07<-gls.modeller(df = age05_18,timesquared = FALSE,timecubed = FALSE,p=6,q=NULL)
fit.08<-gls.modeller(df = age05_18,timesquared = FALSE,timecubed = FALSE,p=8,q=NULL)
fit.09<-gls.modeller(df = age05_18,timesquared = FALSE,timecubed = FALSE,p=12,q=NULL)


fit.10<-gls.modeller(df = age05_18,timesquared = FALSE,timecubed = FALSE,p=NULL,q=1)
fit.11<-gls.modeller(df = age05_18,timesquared = FALSE,timecubed = FALSE,p=NULL,q=2)
fit.12<-gls.modeller(df = age05_18,timesquared = FALSE,timecubed = FALSE,p=NULL,q=3)
fit.13<-gls.modeller(df = age05_18,timesquared = FALSE,timecubed = FALSE,p=NULL,q=4)
fit.14<-gls.modeller(df = age05_18,timesquared = FALSE,timecubed = FALSE,p=NULL,q=6)
fit.15<-gls.modeller(df = age05_18,timesquared = FALSE,timecubed = FALSE,p=NULL,q=8)
#fit.16<-gls.modeller(df = age05_18,timesquared = FALSE,timecubed = FALSE,p = NULL,q=12)
```

fit.16 did not converge.

Compare the AIC values for various possible fits

```
models<-list(fit.01,fit.02,fit.03,fit.04,fit.05,fit.06,fit.07,fit.08,fit.09,fit.10,
             fit.11,fit.12,fit.13,fit.14,fit.15)

age05_18.aictab<-AICcmodavg::aictab(cand.set = models)
```

```
Warning in aictab.AICgls(cand.set = models):
Model names have been supplied automatically in the table
```

```
Warning in aictab.AICgls(cand.set = models):
Check model structure carefully as some models may be redundant
```

```
age05_18.aictab
```

```
Model selection based on AICc:

       K   AICc Delta_AICc AICcWt Cum.Wt      LL
Mod10 11 419.95       0.00   0.21   0.21 -197.44
Mod1  10 420.17       0.22   0.18   0.39 -198.82
Mod2  10 420.17       0.22   0.18   0.58 -198.82
Mod3  11 420.25       0.30   0.18   0.75 -197.59
Mod14 16 421.95       2.00   0.08   0.83 -191.62
Mod11 12 422.37       2.42   0.06   0.89 -197.35
Mod4  12 422.43       2.48   0.06   0.95 -197.38
Mod5  13 424.95       5.00   0.02   0.97 -197.31
Mod12 13 425.03       5.08   0.02   0.98 -197.35
Mod15 18 427.38       7.43   0.01   0.99 -191.36
Mod13 14 427.60       7.65   0.00   0.99 -197.27
Mod6  14 427.67       7.72   0.00   1.00 -197.31
Mod7  16 429.02       9.07   0.00   1.00 -195.15
Mod8  18 432.85      12.90   0.00   1.00 -194.10
Mod9  22 442.09      22.14   0.00   1.00 -192.30
```

Fit.10 is the most appropriate in this model.

Keep the best model and delete the others

```
age05_18.final.gls<-fit.10
rm(fit.01,fit.02,fit.03,fit.04,fit.05,fit.06,fit.07,fit.08,fit.09,fit.10,
   fit.11,fit.12,fit.13,fit.14,fit.15,models)
```

Make table of results

```
age05_18.gls.results.table<-gls.table.maker(age05_18.final.gls)
age05_18.gls.results.table
```

variable	Value	Std.Error	t-value	p-value
(Intercept)	14.18925	0.63043	22.50736	0.00000
time_months	0.05199	0.01868	2.78292	0.00658
announcement.trend	-0.07188	0.05074	-1.41661	0.16009
September	7.34731	0.74194	9.90280	0.00000
October	3.43331	0.74227	4.62544	0.00001
November	3.46429	0.71499	4.84523	0.00001
April	-3.72148	0.68922	-5.39952	0.00000
August	-7.00350	0.71427	-9.80513	0.00000
imp.trend	-0.02827	0.08111	-0.34859	0.72822

Get the fitted values from the model

```
age05_18$predicted<-predict(age05_18.final.gls)

tmp<-as.data.frame(predictSE.gls(mod = age05_18.final.gls,newdata = age05_18))
tmp$ci.min<-tmp$fit-(1.96*tmp$se.fit)
tmp$ci.max<-tmp$fit+(1.96*tmp$se.fit)
tmp<-select(tmp,ci.min,ci.max)

age05_18$ci.min<-tmp$ci.min
age05_18$ci.max<-tmp$ci.max
rm(tmp)
```

Add Counterfactuals 1 and 2 to the data table

```
age05_18$cf<-NA

for(i in 1:nrow(age05_18)){age05_18$cf[i]<-counterfactual.function(i=i,
                                            df = age05_18,
                                            model = age05_18.final.gls)}

age05_18$cf2<-NA

for(i in 1:nrow(age05_18)){age05_18$cf2[i]<-counterfactual2.function(i=i,
                                            df = age05_18,
                                            model = age05_18.final.gls)}
```

Chart the ITS result

```
age05_18.its.chart<-its.model.plot(df = age05_18,model = age05_18.final.gls,points = FALSE
                                   ylim.low = 0,ylim.high = 40, its.title="Ages 5_18y",
                                   its.ytitle = "Incidence rates per 100,000 population" )
age05_18.its.chart
```

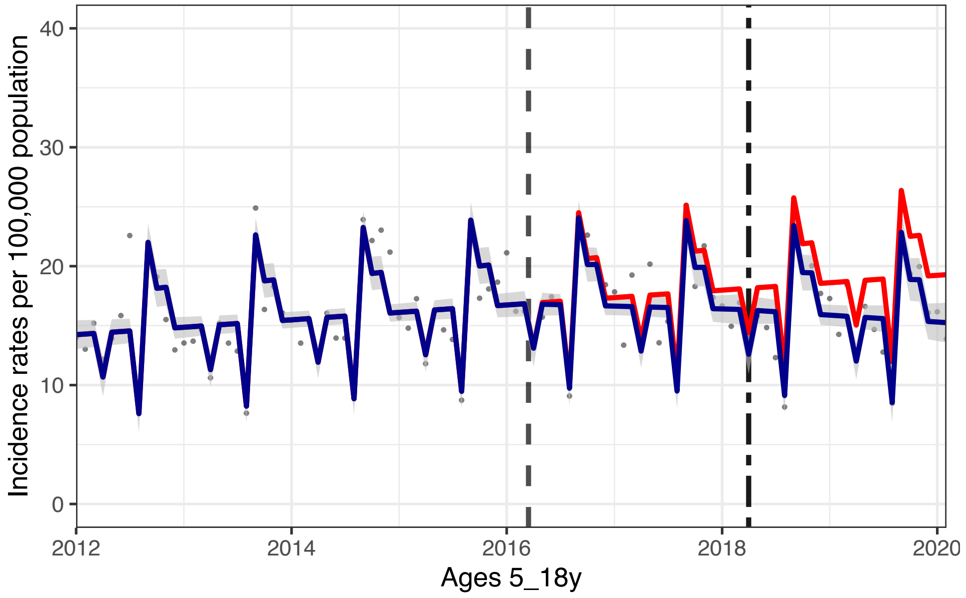

Get values of changes compared to counterfactual 1 (no intervention)

Note: The absolute difference takes no account for the confidence intervals on the counterfactual and is based only on the mean counterfactual values.

```
age05_18.change.absolute <-get.predicted.anyweek(df = age05_18,
                                                  absrel = "abs",pos = 98)
age05_18.change.absolute
```

```
[1] "-4.03, 95% CI -2.36,-5.7) | -20.89%, 95% CI -29.57%,-12.22%)  "
```

Get values of changes compared to counterfactual 2 (no implementation)

Note: The absolute difference takes no account for the confidence intervals on the counterfactual and is based only on the mean counterfactual values.

```
age05_18.change.absolute.cf2 <-get.predicted.anyweek.cf2(df = age05_18,
                                                          absrel = "abs",pos = 98)
age05_18.change.absolute.cf2
```

```
[1] "-0.65, 95% CI 1.02,-2.32) | -4.09%, 95% CI -14.61%,6.43%)  "
```

Calculate mean counts/ 100,000 persons/per month for pre and post-announceent

```
age05_18$phase<-"pre_announcement"
age05_18$phase[which(age05_18$measurement_date>= "2016-03-01")]<-"post_announcement"

age05_18 %>% group_by (phase) %>%
  summarise (mean = mean (admission_prop, na.rm =T),
             (sd = sd (admission_prop, na.rm =T)))
```

```
# A tibble: 2 x 3
  phase              mean `(sd = sd(admission_prop, na.rm = T))`
  <chr>             <dbl>                                   <dbl>
1 post_announcement  16.5                                    3.87
2 pre_announcement   15.8                                    4.04
```

Asthma Analysis (05-09 years)

R code

- Code : Nina Rogers (Nina.Rogers@mrc-epid.cam.ac.uk)
- Code review : Chrissy h. Roberts (chrissy.roberts@LSHTM.ac.uk)

Data Prep and Functions Load

```
source("001_asthma_data_prep_age_sep.R")
source("002_asthma_age_functions.R")
```

Filter the main Age-specific ITS dataset

```
age5_9 <- by_age_asthma %>%
  filter(Age_Group == "B 5-9", measurement_date < "2020-03-01", measurement_date >
  "2011-12-01")
```

Chart admissions data

This provides a simple eyeball test of the data trend across time.

```
  ggplot(age5_9, aes(x=measurement_date, y=admission_prop )) +
  geom_smooth() +
  xlab("")+
    ylab("Admissions per 100,000 population, per month")
```

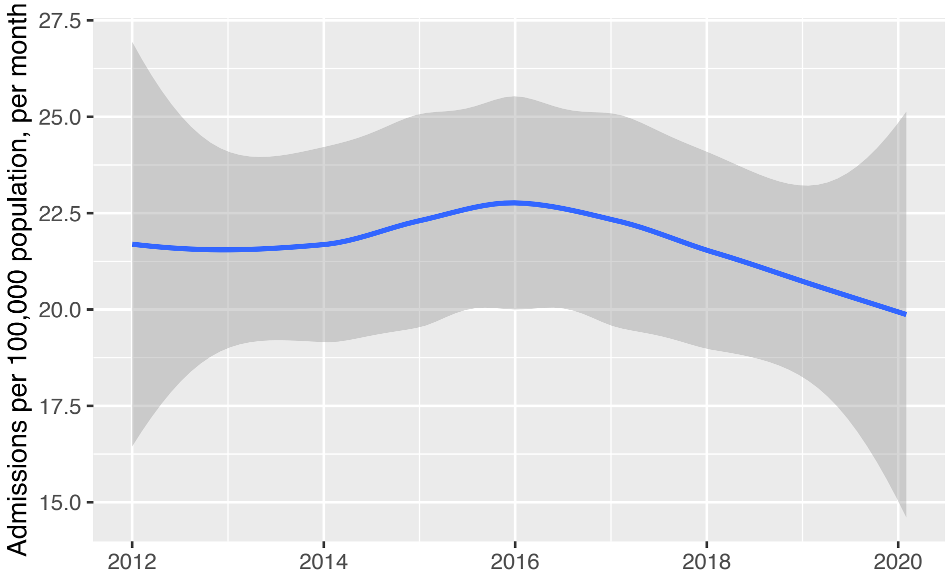

Create the time and intervention variables

This includes definition of time_months (study time), announcement.trend (change in trajectory post announcement) and imp.trend (change in trajectort post implementation).

```
age5_9$time_months <-c(1:98)
age5_9$announcement.trend <- c(rep(0,51),1:47)
age5_9$imp.trend <- c(rep(0,75),1:23)
age5_9 <- age5_9 %>% select(measurement_date, time_months, everything())
```

Run OLS analysis

```
age5_9.ols<-model_ols(df = age5_9)
summary(age5_9.ols)
```

```
Call:
lm(formula = admission_prop ~ time_months + announcement.trend +
    September + October + November + April + August + imp.trend,
    data = df)

Residuals:
    Min      1Q  Median      3Q     Max
-6.2750 -1.8915 -0.0631  1.2984 17.4946

Coefficients:
                    Estimate Std. Error t value Pr(>|t|)
(Intercept)         20.88539    0.98567  21.189  < 2e-16 ***
time_months          0.02162    0.02893   0.747 0.456911
announcement.trend  -0.07557    0.07846  -0.963 0.338070
September           14.47917    1.31626  11.000  < 2e-16 ***
October              5.35581    1.31670   4.068 0.000102 ***
November             5.41750    1.31738   4.112 8.71e-05 ***
April               -5.45796    1.31742  -4.143 7.79e-05 ***
August             -11.90872    1.31603  -9.049 2.97e-14 ***
imp.trend           -0.03145    0.12589  -0.250 0.803308
---
Signif. codes:  0 '***' 0.001 '**' 0.01 '*' 0.05 '.' 0.1 ' ' 1

Residual standard error: 3.489 on 89 degrees of freedom
Multiple R-squared:  0.7647,    Adjusted R-squared:  0.7435
F-statistic: 36.15 on 8 and 89 DF,  p-value: < 2.2e-16
```

Run Durbin-Watson Test

```
age5_9.dwt<-model_dwt(age5_9.ols)
age5_9.dwt
```

```
 lag Autocorrelation D-W Statistic p-value
   1     0.145531583      1.703414   0.072
   2     0.009900350      1.958761   0.668
   3     0.006409952      1.962987   0.836
   4    -0.063061986      2.098222   0.606
   5    -0.114516816      2.199997   0.258
   6    -0.111669234      2.156263   0.310
   7    -0.035513308      1.721083   0.322
   8    -0.078201586      1.769514   0.538
   9    -0.106307118      1.825421   0.784
  10    -0.077810051      1.752084   0.684
  11    -0.056661910      1.688025   0.526
  12    -0.010574066      1.563278   0.138
  13    -0.016813379      1.563745   0.342
  14     0.095833187      1.326615   0.040
  15     0.023523931      1.464808   0.238
  16    -0.013050477      1.529291   0.456
  17     0.009331006      1.482844   0.388
  18    -0.092948453      1.684710   0.932
  19    -0.045908044      1.572943   0.814
  20    -0.006404731      1.465845   0.514
 Alternative hypothesis: rho[lag] != 0
```

Chart acf of ols

```
graph.ols.acf.plots(model = age5_9.ols)
```

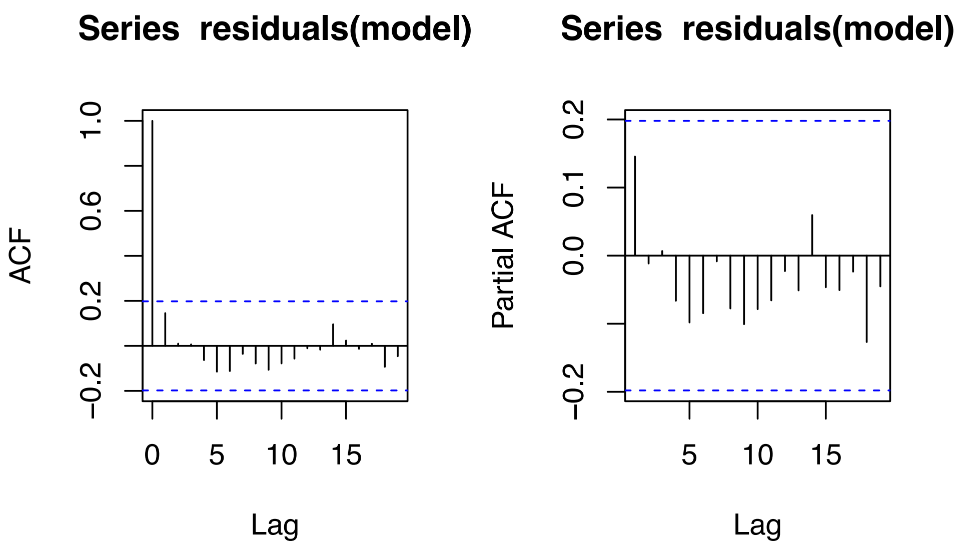

Run gls models with a variety of values of p and q

The DWT and ACF chart suggestlag of 1 may be optimal.

Run a variety of values here to provide belt and braces. Using values 1,2,3,4,6,8 and 12, allows for various cyclical calendar-relevant lags. fit.01 provides a linear relationship between x and y with no lag, whilst fit.02 provides a time-squared function without lag.

```
fit.01<-gls.modeller(df= age5_9,timesquared= FALSE,timecubed= FALSE,p=NULL,q=NULL)
fit.02<-gls.modeller(df= age5_9,timesquared= TRUE,timecubed= FALSE,p=NULL,q=NULL)

fit.03<-gls.modeller(df= age5_9,timesquared= FALSE,timecubed= FALSE,p=1,q=NULL)
fit.04<-gls.modeller(df= age5_9,timesquared= FALSE,timecubed= FALSE,p=2,q=NULL)
fit.05<-gls.modeller(df= age5_9,timesquared= FALSE,timecubed= FALSE,p=3,q=NULL)
fit.06<-gls.modeller(df= age5_9,timesquared= FALSE,timecubed= FALSE,p=4,q=NULL)
fit.07<-gls.modeller(df= age5_9,timesquared= FALSE,timecubed= FALSE,p=6,q=NULL)
fit.08<-gls.modeller(df= age5_9,timesquared= FALSE,timecubed= FALSE,p=8,q=NULL)
fit.09<-gls.modeller(df= age5_9,timesquared= FALSE,timecubed= FALSE,p=12,q=NULL)

fit.10<-gls.modeller(df= age5_9,timesquared= FALSE,timecubed= FALSE,p=NULL,q=1)
fit.11<-gls.modeller(df= age5_9,timesquared= FALSE,timecubed= FALSE,p=NULL,q=2)
fit.12<-gls.modeller(df= age5_9,timesquared= FALSE,timecubed= FALSE,p=NULL,q=3)
fit.13<-gls.modeller(df= age5_9,timesquared= FALSE,timecubed= FALSE,p=NULL,q=4)
fit.14<-gls.modeller(df= age5_9,timesquared= FALSE,timecubed= FALSE,p=NULL,q=6)
fit.15<-gls.modeller(df= age5_9,timesquared= FALSE,timecubed= FALSE,p=NULL,q=8)
fit.16<-gls.modeller(df= age5_9,timesquared= FALSE,timecubed= FALSE,p=NULL,q=12)
```

Compare the AIC values for various possible fits

```
models<-list(fit.01,fit.02,fit.03,fit.04,fit.05,fit.06,fit.07,fit.08,fit.09,fit.10,
             fit.11,fit.12,fit.13,fit.14,fit.15,fit.16)

age5_9.aictab<-AICcmodavg::aictab(cand.set = models)
```

```
Warning in aictab.AICgls(cand.set = models):
Model names have been supplied automatically in the table

Warning in aictab.AICgls(cand.set = models):
Check model structure carefully as some models may be redundant
```

```
age5_9.aictab
```

```
Model selection based on AICc:

       K   AICc Delta_AICc AICcWt Cum.Wt      LL
Mod1  10 536.11       0.00   0.21   0.21 -256.79
Mod2  10 536.11       0.00   0.21   0.43 -256.79
Mod10 11 536.26       0.14   0.20   0.62 -255.59
Mod3  11 536.36       0.24   0.19   0.81 -255.64
Mod11 12 538.84       2.73   0.05   0.87 -255.59
Mod4  12 538.86       2.74   0.05   0.92 -255.59
Mod14 16 540.82       4.71   0.02   0.94 -251.05
Mod15 18 540.91       4.80   0.02   0.96 -248.13
Mod12 13 541.50       5.38   0.01   0.98 -255.58
Mod5  13 541.52       5.41   0.01   0.99 -255.59
Mod6  14 543.83       7.72   0.00   1.00 -255.39
Mod13 14 544.09       7.98   0.00   1.00 -255.52
Mod7  16 547.82      11.71   0.00   1.00 -254.55
Mod8  18 549.52      13.40   0.00   1.00 -252.43
Mod16 22 550.83      14.71   0.00   1.00 -246.67
Mod9  22 557.44      21.32   0.00   1.00 -249.97
```

Fit.01, the model with no lag is the most appropriate in this model.

Keep the best model and delete the others

```
age5_9.final.gls<-fit.01
rm(fit.01,fit.02,fit.03,fit.04,fit.05,fit.06,fit.07,fit.08,fit.09,fit.10,
   fit.11,fit.12,fit.13,fit.14,fit.15,fit.16,models)
```

Make table of results

```
age5_9.gls.results.table<-gls.table.maker(age5_9.final.gls)
age5_9.gls.results.table
```

variable	Value	Std.Error	t-value	p-value
(Intercept)	20.88539	0.98567	21.18913	0.00000
time_months	0.02162	0.02893	0.74720	0.45691
announcement.trend	-0.07557	0.07846	-0.96317	0.33807
September	14.47917	1.31626	11.00027	0.00000
October	5.35581	1.31670	4.06759	0.00010
November	5.41750	1.31738	4.11234	0.00009
April	-5.45796	1.31742	-4.14293	0.00008
August	-11.90872	1.31603	-9.04894	0.00000
imp.trend	-0.03145	0.12589	-0.24981	0.80331

Get the fitted values from the model

```
age5_9$predicted<-predict(age5_9.final.gls)

tmp<-as.data.frame(predictSE.gls(mod = age5_9.final.gls,newdata = age5_9))
tmp$ci.min<-tmp$fit-(1.96*tmp$se.fit)
tmp$ci.max<-tmp$fit+(1.96*tmp$se.fit)
tmp<-select(tmp,ci.min,ci.max)

age5_9$ci.min<-tmp$ci.min
age5_9$ci.max<-tmp$ci.max
rm(tmp)
```

Add Counterfactuals 1 and 2 to the data table

```
age5_9$cf<-NA

for(i in 1:nrow(age5_9)){age5_9$cf[i]<-counterfactual.function(i=i,
                                              df = age5_9,
                                              model = age5_9.final.gls)}

age5_9$cf2<-NA

for(i in 1:nrow(age5_9)){age5_9$cf2[i]<-counterfactual2.function(i=i,
                                              df = age5_9,
                                              model = age5_9.final.gls)}
```

Chart the ITS result

```
age5_9.its.chart<-its.model.plot(df = age5_9,model = age5_9.final.gls,points = FALSE,
                                 ylim.low = 0,ylim.high = 40,
                                 its.title="Ages 5_9y",
                                 its.ytitle = "Incidence rates per 100,000 population" )
age5_9.its.chart
```

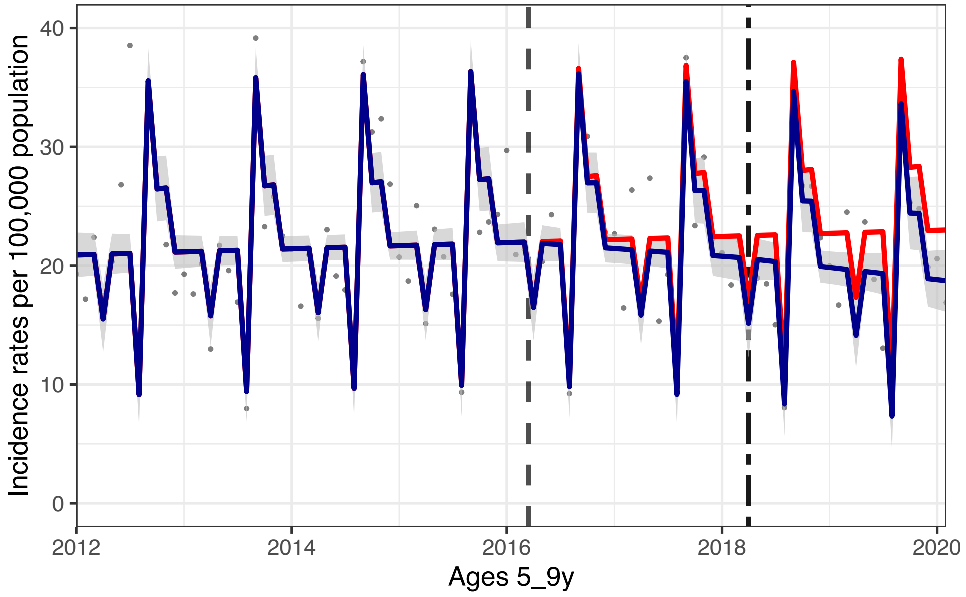

Get values of changes compared to counterfactual 1 (no intervention)

Note: The absolute difference takes no account for the confidence intervals on the counterfactual and is based only on the mean counterfactual values.

```
age5_9.change.absolute <-get.predicted.anyweek(df = age5_9,
                                               absrel = "abs",pos = 98)
age5_9.change.absolute
```

```
[1] "-4.27, 95% CI -1.65,-6.9) | -18.58%, 95% CI -29.97%,-7.19%)  "
```

Get values of changes compared to counterfactual 2 (no implementation)

Note: The absolute difference takes no account for the confidence intervals on the counterfactual and is based only on the mean counterfactual values.

```
age5_9.change.absolute.cf2 <-get.predicted.anyweek.cf2(df = age5_9,
                                                       absrel = "abs",pos = 98)
age5_9.change.absolute.cf2
```

```
[1] "-0.72, 95% CI 1.9,-3.34) | -3.72%, 95% CI -17.19%,9.75%)  "
```

Calculate mean counts/ 100,000 persons/per month for pre and post-announceent

```
age5_9$phase<-"pre_announcement"
age5_9$phase[which(age5_9$measurement_date>= "2016-03-01")]<-"post_announcement"

age5_9 %>% group_by (phase) %>%
  summarise (mean = mean (admission_prop, na.rm =T),
             (sd = sd (admission_prop, na.rm =T)))
```

```
# A tibble: 2 x 3
  phase              mean `(sd = sd(admission_prop, na.rm = T))`
  <chr>             <dbl>                                    <dbl>
1 post_announcement  21.3                                     6.69
2 pre_announcement   22.0                                     7.13
```

Asthma Analysis (10-14 years)

R code

- Code : Nina Rogers (Nina.Rogers@mrc-epid.cam.ac.uk)
- Code review : Chrissy h. Roberts (chrissy.roberts@LSHTM.ac.uk)

Data Prep and Functions Load

```
source("001_asthma_data_prep_age_sep.R")
source("002_asthma_age_functions.R")
```

Filter the main Age-specific ITS dataset

```
age10_14 <- by_age_asthma %>%
  filter(Age_Group == "C 10-14", measurement_date < "2020-03-01",
         measurement_date > "2011-12-01")
```

Chart admissions data

This provides a simple eyeball test of the data trend across time.

```
ggplot(age10_14, aes(x=measurement_date, y=admission_prop )) +
geom_smooth() +
xlab("")+
  ylab("Admissions per 100,000 population, per month")
```

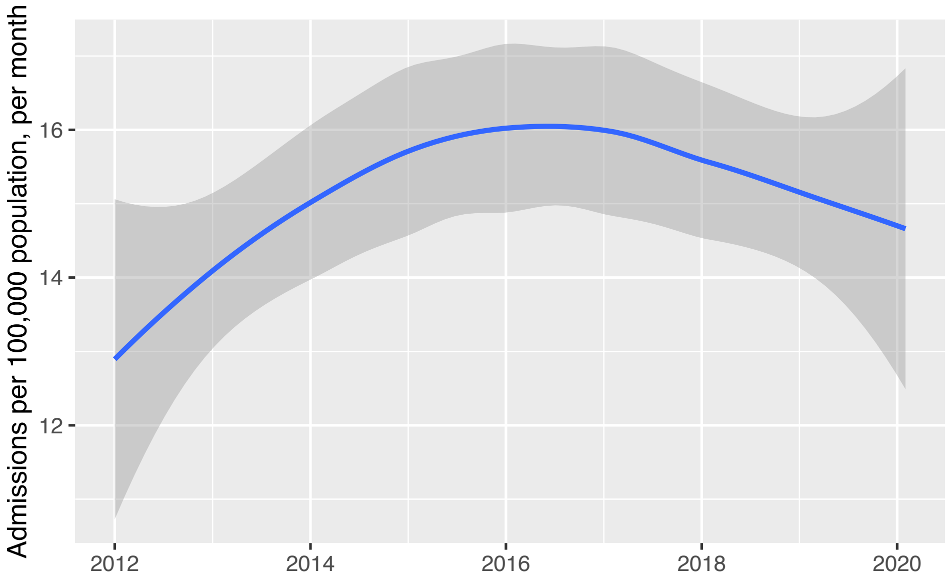

Create the time and intervention variables

This includes definition of time_months (study time), announcement.trend (change in trajectory post announcement) and imp.trend (change in trajectort post implementation).

```
age10_14$time_months <-c(1:98)
age10_14$announcement.trend <- c(rep(0,51),1:47)
age10_14$imp.trend <- c(rep(0,75),1:23)
age10_14 <- age10_14 %>% select(measurement_date, time_months, everything())
```

Run OLS analysis

```
age10_14.ols<-model_ols(df = age10_14)
summary(age10_14.ols)
```

```
Call:
lm(formula = admission_prop ~ time_months + announcement.trend +
    September + October + November + April + August + imp.trend,
    data = df)

Residuals:
    Min      1Q  Median      3Q     Max
-3.2244 -1.1223 -0.1075  0.6317  5.4412

Coefficients:
                    Estimate Std. Error t value Pr(>|t|)
(Intercept)        13.407657   0.448256  29.911  < 2e-16 ***
time_months         0.058668   0.013157   4.459 2.39e-05 ***
announcement.trend -0.101707   0.035679  -2.851  0.00542 **
September           3.632376   0.598600   6.068 3.09e-08 ***
October             1.790273   0.598803   2.990  0.00361 **
November            3.142340   0.599110   5.245 1.05e-06 ***
April              -2.797019   0.599127  -4.668 1.07e-05 ***
August             -5.934153   0.598499  -9.915 4.80e-16 ***
imp.trend           0.007457   0.057251   0.130  0.89666
---
Signif. codes:  0 '***' 0.001 '**' 0.01 '*' 0.05 '.' 0.1 ' ' 1

Residual standard error: 1.587 on 89 degrees of freedom
Multiple R-squared:  0.7332,    Adjusted R-squared:  0.7092
F-statistic: 30.57 on 8 and 89 DF,  p-value: < 2.2e-16
```

Run Durbin-Watson Test

```
age10_14.dwt<-model_dwt(age10_14.ols)
age10_14.dwt
```

```
 lag Autocorrelation D-W Statistic p-value
   1     0.224909323      1.541080   0.008
   2     0.082124879      1.825234   0.292
   3    -0.007561328      1.998996   0.994
   4    -0.039311068      2.059449   0.766
   5    -0.116043774      2.211700   0.226
   6    -0.209678568      2.386907   0.020
   7    -0.075395511      2.091683   0.444
   8    -0.117366534      2.154739   0.206
   9    -0.059898941      2.009048   0.462
  10    -0.028557707      1.924267   0.730
  11     0.002069300      1.825890   0.968
  12     0.038752138      1.708229   0.320
  13     0.037257834      1.704263   0.768
  14    -0.045034722      1.853306   0.592
  15    -0.035368219      1.818880   0.666
  16    -0.059048473      1.865845   0.418
  17    -0.075647987      1.892076   0.302
  18    -0.196808220      2.121140   0.038
  19    -0.122269811      1.953598   0.128
  20    -0.000197071      1.662875   0.778
 Alternative hypothesis: rho[lag] != 0
```

Chart acf of ols

```
graph.ols.acf.plots(model = age10_14.ols)
```

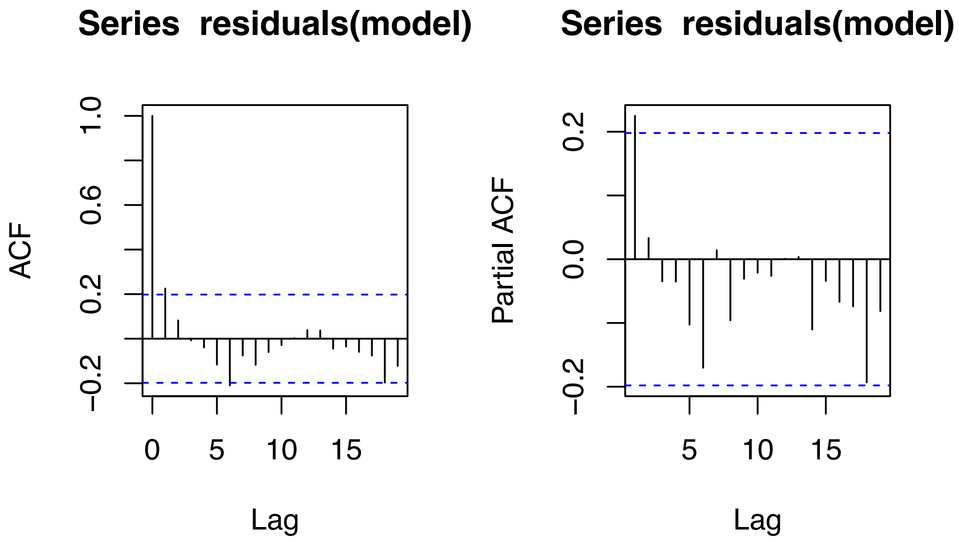

Run gls models with a variety of values of p and q

The DWT and ACF chart suggestlag of 1 may be optimal.

Run a variety of values here to provide belt and braces. Using values 1,2,3,4,6,8 and 12, allows for various cyclical calendar-relevant lags. fit.01 provides a linear relationship between x and y with no lag, whilst fit.02 provides a time-squared function without lag.

```
fit.01<-gls.modeller(df = age10_14,timesquared = FALSE,timecubed = FALSE,p=NULL,q=NULL)
fit.02<-gls.modeller(df = age10_14,timesquared = TRUE,timecubed = FALSE,p=NULL,q=NULL)

fit.03<-gls.modeller(df = age10_14,timesquared = FALSE,timecubed = FALSE,p=1,q=NULL)
fit.04<-gls.modeller(df = age10_14,timesquared = FALSE,timecubed = FALSE,p=2,q=NULL)
fit.05<-gls.modeller(df = age10_14,timesquared = FALSE,timecubed = FALSE,p=3,q=NULL)
fit.06<-gls.modeller(df = age10_14,timesquared = FALSE,timecubed = FALSE,p=4,q=NULL)
fit.07<-gls.modeller(df = age10_14,timesquared = FALSE,timecubed = FALSE,p=6,q=NULL)
fit.08<-gls.modeller(df = age10_14,timesquared = FALSE,timecubed = FALSE,p=8,q=NULL)
fit.09<-gls.modeller(df = age10_14,timesquared = FALSE,timecubed = FALSE,p=12,q=NULL)

fit.10<-gls.modeller(df = age10_14,timesquared = FALSE,timecubed = FALSE,p=NULL,q=1)
fit.11<-gls.modeller(df = age10_14,timesquared = FALSE,timecubed = FALSE,p=NULL,q=2)
fit.12<-gls.modeller(df = age10_14,timesquared = FALSE,timecubed = FALSE,p=NULL,q=3)
fit.13<-gls.modeller(df = age10_14,timesquared = FALSE,timecubed = FALSE,p=NULL,q=4)
fit.14<-gls.modeller(df = age10_14,timesquared = FALSE,timecubed = FALSE,p=NULL,q=6)
#fit.15<-gls.modeller(df = age10_14,timesquared = FALSE,timecubed = FALSE,p = NULL,q=8)
fit.16<-gls.modeller(df = age10_14,timesquared = FALSE,timecubed = FALSE,p=NULL,q=12)
```

NB fit.15 failed to converge

Compare the AIC values for various possible fits

```
models<-list(fit.01,fit.02,fit.03,fit.04,fit.05,fit.06,fit.07,fit.08,fit.09,fit.10,
             fit.11,fit.12,fit.13,fit.14,fit.16)

age10_14.aictab<-AICcmodavg::aictab(cand.set = models)
```

```
Warning in aictab.AICgls(cand.set = models):
Model names have been supplied automatically in the table

Warning in aictab.AICgls(cand.set = models):
Check model structure carefully as some models may be redundant
```

```
age10_14.aictab
```

```
Model selection based on AICc:

       K   AICc Delta_AICc AICcWt Cum.Wt      LL
Mod3  11 378.43       0.00   0.33   0.33 -176.68
Mod10 11 379.12       0.69   0.23   0.56 -177.02
Mod11 12 380.68       2.25   0.11   0.66 -176.51
Mod4  12 380.94       2.51   0.09   0.75 -176.63
Mod1  10 381.67       3.24   0.06   0.82 -179.57
Mod2  10 381.67       3.24   0.06   0.88 -179.57
Mod14 16 382.71       4.28   0.04   0.92 -172.00
Mod5  13 383.16       4.72   0.03   0.95 -176.41
Mod12 13 383.34       4.91   0.03   0.98 -176.50
Mod6  14 385.39       6.96   0.01   0.99 -176.17
Mod13 14 386.07       7.63   0.01   1.00 -176.50
Mod7  16 388.04       9.61   0.00   1.00 -174.66
Mod15 22 391.17      12.74   0.00   1.00 -166.84
Mod8  18 392.19      13.75   0.00   1.00 -173.76
Mod9  22 400.40      21.97   0.00   1.00 -171.45
```

Fit.03, the model with p=1 is the most appropriate in this model.

Keep the best model and delete the others

```
age10_14.final.gls<-fit.03
rm(fit.01,fit.02,fit.03,fit.04,fit.05,fit.06,fit.07,fit.08,fit.09,fit.10,
   fit.11,fit.12,fit.13,fit.14,fit.16,models)
```

Make table of results

```
age10_14.gls.results.table<-gls.table.maker(age10_14.final.gls)
age10_14.gls.results.table
```

variable	Value	Std.Error	t-value	p-value
(Intercept)	13.44766	0.56766	23.68973	0.00000
time_months	0.05860	0.01693	3.46132	0.00083
announcement.trend	-0.10051	0.04605	-2.18284	0.03168
September	3.63156	0.60728	5.98001	0.00000
October	1.68040	0.60750	2.76611	0.00690
November	2.75873	0.57587	4.79051	0.00001
April	-2.98423	0.54417	-5.48401	0.00000
August	-5.74941	0.57546	-9.99106	0.00000
imp.trend	0.00237	0.07329	0.03233	0.97428

Get the fitted values from the model

```
age10_14$predicted<-predict(age10_14.final.gls)

tmp<-as.data.frame(predictSE.gls(mod = age10_14.final.gls,newdata = age10_14))
tmp$ci.min<-tmp$fit-(1.96*tmp$se.fit)
tmp$ci.max<-tmp$fit+(1.96*tmp$se.fit)
tmp<-select(tmp,ci.min,ci.max)

age10_14$ci.min<-tmp$ci.min
age10_14$ci.max<-tmp$ci.max
rm(tmp)
```

Add Counterfactuals 1 and 2 to the data table

```
age10_14$cf<-NA

for(i in 1:nrow(age10_14)){age10_14$cf[i]<-counterfactual.function(i=i,
                                                  df = age10_14,
                                                  model = age10_14.final.gls)}

age10_14$cf2<-NA

for(i in 1:nrow(age10_14)){age10_14$cf2[i]<-counterfactual2.function(i=i,
                                                  df = age10_14,
                                                  model = age10_14.final.gls)}
```

Chart the ITS result

```
age10_14.its.chart<-its.model.plot(df = age10_14,
                                   model = age10_14.final.gls,
                                   points = FALSE,
                                   ylim.low = 0, ylim.high = 40,
                                   its.title="Ages 10_14y",
                                   its.ytitle = "Incidence rates per 100,000 population" )
age10_14.its.chart
```

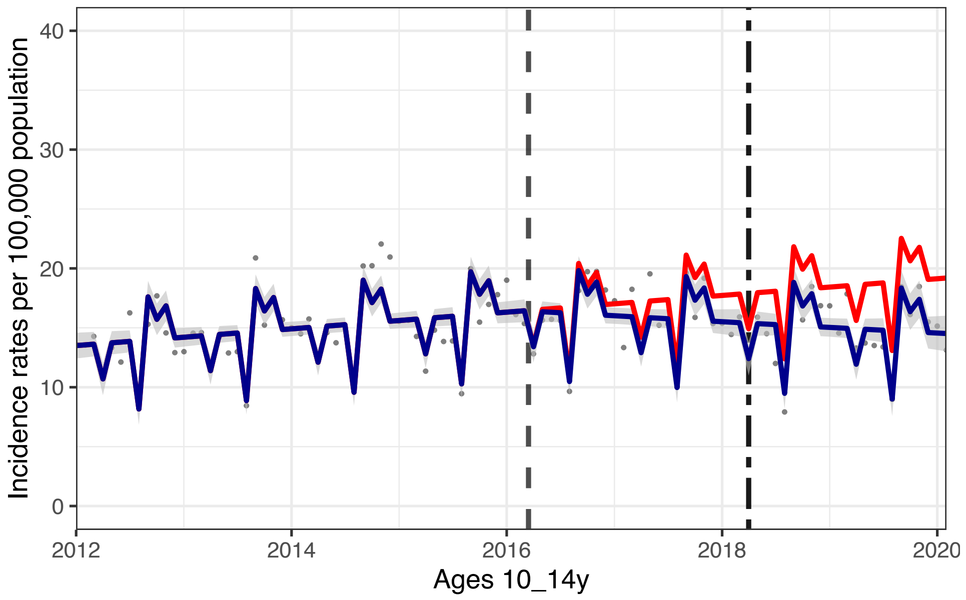

Get values of changes compared to counterfactual 1 (no intervention)

Note: The absolute difference takes no account for the confidence intervals on the counterfactual and is based only on the mean counterfactual values.

```
age10_14.change.absolute <-get.predicted.anyweek(df = age10_14,
                                                  absrel = "abs",pos = 98)
age10_14.change.absolute
```

```
[1] "-4.67, 95% CI -3.17,-6.17) | -24.33%, 95% CI -32.14%,-16.52%)  "
```

Get values of changes compared to counterfactual 2 (no implementation)

Note: The absolute difference takes no account for the confidence intervals on the counterfactual and is based only on the mean counterfactual values.

```
age10_14.change.absolute.cf2 <-get.predicted.anyweek.cf2(df = age10_14,
                                                          absrel = "abs",pos = 98)
age10_14.change.absolute.cf2
```

```
[1] "0.05, 95% CI 1.55,-1.44) | 0.38%, 95% CI -9.98%,10.74%)  "
```

Calculate mean counts/ 100,000 persons/per month for pre and post-announceent

```
age10_14$phase<-"pre_announcement"
age10_14$phase[which(age10_14$measurement_date>= "2016-03-01")]<-"post_announcement"

age10_14 %>% group_by (phase) %>%
  summarise (mean = mean (admission_prop, na.rm =T),
             (sd = sd (admission_prop, na.rm =T)))
```

```
# A tibble: 2 x 3
  phase              mean `(sd = sd(admission_prop, na.rm = T))`
  <chr>             <dbl>                                    <dbl>
1 post_announcement  15.4                                     2.83
2 pre_announcement   14.9                                     3.05
```

Asthma Analysis (15-18 years)

R code

- Code : Nina Rogers (Nina.Rogers@mrc-epid.cam.ac.uk)
- Code review : Chrissy h. Roberts (chrissy.roberts@LSHTM.ac.uk)

Data Prep and Functions Load

```
source("001_asthma_data_prep_age_sep.R")
source("002_asthma_age_functions.R")
```

Filter the main Age-specific ITS dataset

```
age15_18 <- by_age_asthma %>%
  filter(Age_Group == "D 15-18", measurement_date < "2020-03-01",
         measurement_date > "2011-12-01")
```

Chart admissions data

This provides a simple eyeball test of the data trend across time.

```
  ggplot(age15_18, aes(x=measurement_date, y=admission_prop )) +
  geom_smooth() +
  xlab("")+
    ylab("Admissions per 100,000 population, per month")
```

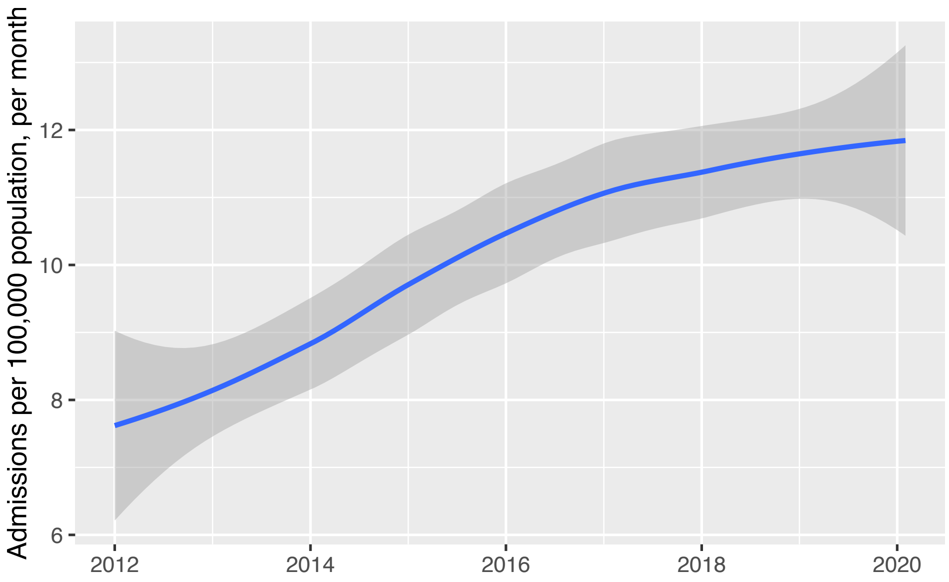

Create the time and intervention variables

This includes definition of time_months (study time), announcement.trend (change in trajectory post announcement) and imp.trend (change in trajectort post implementation).

```
age15_18$time_months <-c(1:98)
age15_18$announcement.trend <- c(rep(0,51),1:47)
age15_18$imp.trend <- c(rep(0,75),1:23)
age15_18 <- age15_18 %>% select(measurement_date, time_months, everything())
```

Run OLS analysis

```
age15_18.ols<-model_ols(df = age15_18)
summary(age15_18.ols)
```

```
Call:
lm(formula = admission_prop ~ time_months + announcement.trend +
    September + October + November + April + August + imp.trend,
    data = df)

Residuals:
    Min      1Q  Median      3Q     Max
-2.4390 -0.7085 -0.1064  0.5344  4.8410

Coefficients:
                    Estimate Std. Error t value Pr(>|t|)
(Intercept)         7.096268   0.338223  20.981  < 2e-16 ***
time_months         0.059412   0.009927   5.985 4.46e-08 ***
announcement.trend -0.015966   0.026921  -0.593  0.55465
September           2.605103   0.451662   5.768 1.15e-07 ***
October             3.093726   0.451816   6.847 9.33e-10 ***
November            2.187088   0.452047   4.838 5.46e-06 ***
April              -1.283786   0.452060  -2.840  0.00559 **
August             -2.018938   0.451586  -4.471 2.29e-05 ***
imp.trend          -0.040503   0.043198  -0.938  0.35098
---
Signif. codes:  0 '***' 0.001 '**' 0.01 '*' 0.05 '.' 0.1 ' ' 1

Residual standard error: 1.197 on 89 degrees of freedom
Multiple R-squared:  0.7535,    Adjusted R-squared:  0.7314
F-statistic: 34.01 on 8 and 89 DF,  p-value: < 2.2e-16
```

Run Durbin-Watson Test

```
age15_18.dwt<-model_dwt(age15_18.ols)
age15_18.dwt
```

```
 lag Autocorrelation D-W Statistic p-value
   1      0.16510213      1.658800   0.056
   2     -0.09494116      2.177968   0.460
   3      0.05645634      1.872195   0.492
   4      0.05299001      1.852721   0.534
   5     -0.21688311      2.384608   0.032
   6     -0.36018247      2.669665   0.000
   7     -0.18090146      2.218854   0.162
   8     -0.06649618      1.988219   0.690
   9      0.10806590      1.603085   0.190
  10      0.00455941      1.801699   0.748
  11      0.09663284      1.608709   0.270
  12      0.20366912      1.387929   0.012
  13      0.22516469      1.336040   0.034
  14     -0.11209904      1.952055   0.310
  15     -0.02949378      1.780002   0.834
  16     -0.04850049      1.811883   0.584
  17     -0.16012929      2.034748   0.114
  18     -0.26881892      2.237903   0.006
  19     -0.09152985      1.878956   0.254
  20      0.05843328      1.534693   0.744
 Alternative hypothesis: rho[lag] != 0
```

Chart acf of ols

```
graph.ols.acf.plots(model = age15_18.ols)
```

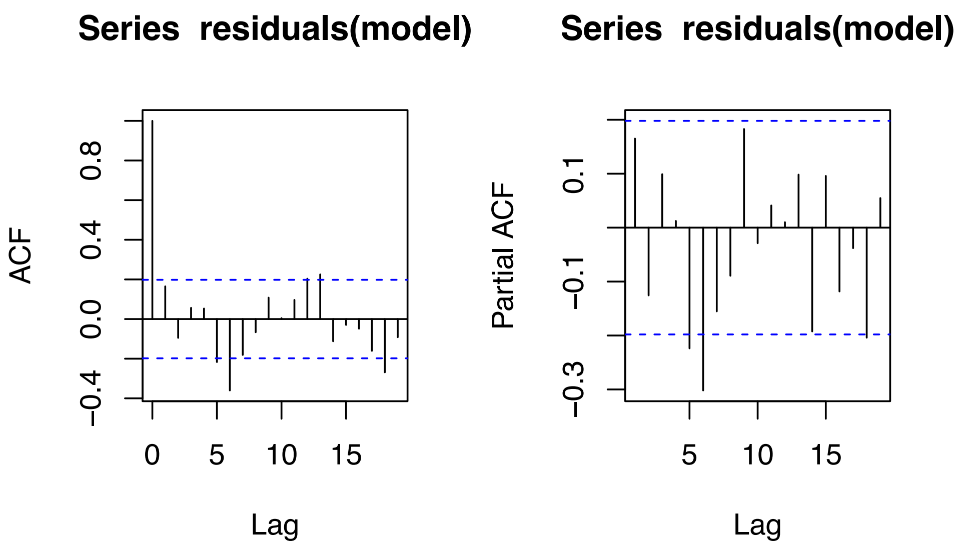

Run gls models with a variety of values of p and q

The DWT and ACF chart suggestlag of 1 may be optimal.

Run a variety of values here to provide belt and braces. Using values 1,2,3,4,6,8 and 12, allows for various cyclical calendar-relevant lags. fit.01 provides a linear relationship between x and y with no lag, whilst fit.02 provides a time-squared function without lag.

```
fit.01<-gls.modeller(df = age15_18,timesquared = FALSE,timecubed = FALSE,p=NULL,q=NULL)
fit.02<-gls.modeller(df = age15_18,timesquared = TRUE,timecubed = FALSE,p=NULL,q=NULL)

fit.03<-gls.modeller(df = age15_18,timesquared = FALSE,timecubed = FALSE,p=1,q=NULL)
fit.04<-gls.modeller(df = age15_18,timesquared = FALSE,timecubed = FALSE,p=2,q=NULL)
fit.05<-gls.modeller(df = age15_18,timesquared = FALSE,timecubed = FALSE,p=3,q=NULL)
fit.06<-gls.modeller(df = age15_18,timesquared = FALSE,timecubed = FALSE,p=4,q=NULL)
fit.07<-gls.modeller(df = age15_18,timesquared = FALSE,timecubed = FALSE,p=6,q=NULL)
fit.08<-gls.modeller(df = age15_18,timesquared = FALSE,timecubed = FALSE,p=8,q=NULL)
fit.09<-gls.modeller(df = age15_18,timesquared = FALSE,timecubed = FALSE,p=12,q=NULL)


fit.10<-gls.modeller(df = age15_18,timesquared = FALSE,timecubed = FALSE,p=NULL,q=1)
fit.11<-gls.modeller(df = age15_18,timesquared = FALSE,timecubed = FALSE,p=NULL,q=2)
fit.12<-gls.modeller(df = age15_18,timesquared = FALSE,timecubed = FALSE,p=NULL,q=3)
fit.13<-gls.modeller(df = age15_18,timesquared = FALSE,timecubed = FALSE,p=NULL,q=4)
fit.14<-gls.modeller(df = age15_18,timesquared = FALSE,timecubed = FALSE,p=5,q=NULL)
fit.15<-gls.modeller(df = age15_18,timesquared = FALSE,timecubed = FALSE,p=NULL,q=8)
fit.16<-gls.modeller(df = age15_18,timesquared = FALSE,timecubed = FALSE,p=18,q=NULL)
```

fit.14 and fit.16 did not converge. Switched fit.14 from default q=6 to p=5 to checkbasedon acf chart if p-5 is a good model

Compare the AIC values for various possible fits

```
models<-list(fit.01,fit.02,fit.03,fit.04,fit.05,fit.06,fit.07,fit.08,fit.09,fit.10,
             fit.11,fit.12,fit.13,fit.14,fit.15,fit.16)

age15_18.aictab<-AICcmodavg::aictab(cand.set = models)
```

```
Warning in aictab.AICgls(cand.set = models):
Model names have been supplied automatically in the table
```

```
Warning in aictab.AICgls(cand.set = models):
Check model structure carefully as some models may be redundant
```

```
age15_18.aictab
```

```
Model selection based on AICc:

       K   AICc Delta_AICc AICcWt Cum.Wt      LL
Mod15 18 312.13       0.00   0.94   0.94 -133.74
Mod7  16 318.73       6.59   0.03   0.98 -140.00
Mod8  18 320.38       8.24   0.02   0.99 -137.86
Mod10 11 324.79      12.66   0.00   0.99 -149.86
Mod3  11 325.79      13.66   0.00   0.99 -150.36
Mod11 12 326.43      14.29   0.00   1.00 -149.38
Mod14 15 326.43      14.30   0.00   1.00 -145.29
Mod1  10 326.47      14.33   0.00   1.00 -151.97
Mod2  10 326.47      14.33   0.00   1.00 -151.97
Mod4  12 326.67      14.54   0.00   1.00 -149.50
Mod13 14 327.34      15.21   0.00   1.00 -147.14
Mod5  13 327.94      15.80   0.00   1.00 -148.80
Mod12 13 329.02      16.89   0.00   1.00 -149.35
Mod9  22 329.17      17.04   0.00   1.00 -135.84
Mod6  14 329.62      17.49   0.00   1.00 -148.28
Mod16 28 330.27      18.13   0.00   1.00 -125.37
```

Fit.15 is the most appropriate in this model.

Keep the best model and delete the others

```
age15_18.final.gls<-fit.15
rm(fit.01,fit.02,fit.03,fit.04,fit.05,fit.06,fit.07,fit.08,fit.09,fit.10,
   fit.11,fit.12,fit.13,fit.14,fit.15,fit.16,models)
```

Make table of results

```
age15_18.gls.results.table<-gls.table.maker(age15_18.final.gls)
age15_18.gls.results.table
```

variable	Value	Std.Error	t-value	p-value
(Intercept)	7.12733	0.15409	46.25372	0.00000
time_months	0.06301	0.00464	13.57937	0.00000
announcement.trend	-0.01721	0.01385	-1.24288	0.21718
September	2.00535	0.11531	17.39130	0.00000
October	2.92605	0.38712	7.55854	0.00000
November	1.64596	0.31663	5.19831	0.00000
April	-1.54298	0.38535	-4.00410	0.00013
August	-2.65206	0.31613	-8.38909	0.00000
imp.trend	-0.05930	0.02753	-2.15373	0.03396

Get the fitted values from the model

```
age15_18$predicted<-predict(age15_18.final.gls)

tmp<-as.data.frame(predictSE.gls(mod = age15_18.final.gls,newdata = age15_18))
tmp$ci.min<-tmp$fit-(1.96*tmp$se.fit)
tmp$ci.max<-tmp$fit+(1.96*tmp$se.fit)
tmp<-select(tmp,ci.min,ci.max)

age15_18$ci.min<-tmp$ci.min
age15_18$ci.max<-tmp$ci.max
rm(tmp)
```

Add Counterfactuals 1 and 2 to the data table

```
age15_18$cf<-NA

for(i in 1:nrow(age15_18)){age15_18$cf[i]<-counterfactual.function(i=i,
                                                df = age15_18,
                                                model = age15_18.final.gls)}

age15_18$cf2<-NA

for(i in 1:nrow(age15_18)){age15_18$cf2[i]<-counterfactual2.function(i=i,
                                                df = age15_18,
                                                model = age15_18.final.gls)}
```

Chart the ITS result

```
age15_18.its.chart<-its.model.plot(df = age15_18,
                                    model = age15_18.final.gls,
                                    points = FALSE,
                                    ylim.low = 0, ylim.high = 40,
                                    its.title="Ages 15_18y",
                                    its.ytitle = "Incidence rates per 100,000 population" )
age15_18.its.chart
```

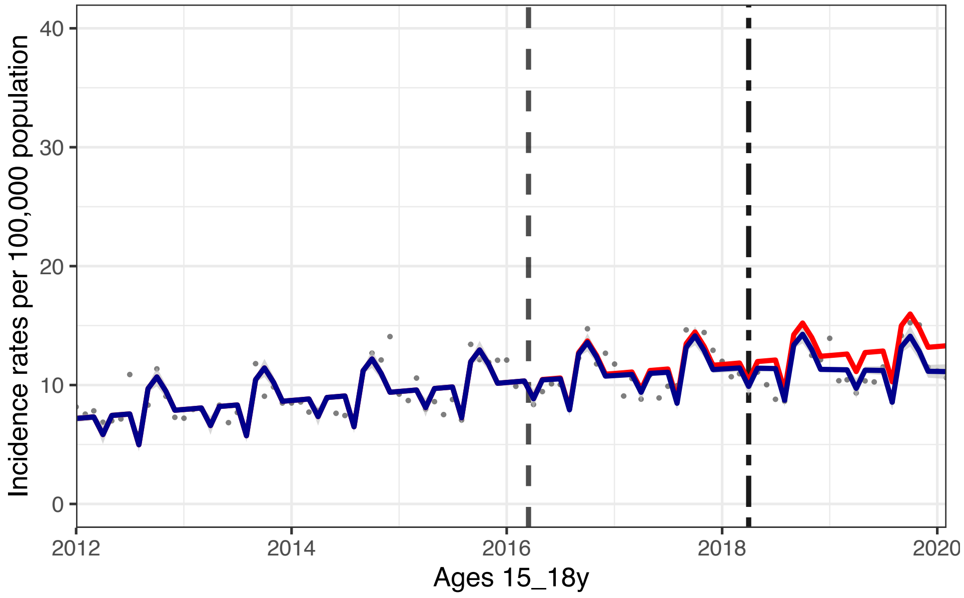

Get values of changes compared to counterfactual 1 (no intervention)

Note: The absolute difference takes no account for the confidence intervals on the counterfactual and is based only on the mean counterfactual values.

```
age15_18.change.absolute <-get.predicted.anyweek(df = age15_18,
                                                  absrel = "abs",pos = 98)
age15_18.change.absolute
```

```
[1] "-2.17, 95% CI -1.56,-2.79) | -16.33%, 95% CI -20.94%,-11.73%)  "
```

Get values of changes compared to counterfactual 2 (no implementation)

Note: The absolute difference takes no account for the confidence intervals on the counterfactual and is based only on the mean counterfactual values.

```
age15_18.change.absolute.cf2 <-get.predicted.anyweek.cf2(df = age15_18,
                                                          absrel = "abs",pos = 98)
age15_18.change.absolute.cf2
```

```
[1] "-1.36, 95% CI -0.75,-1.98) | -10.92%, 95% CI -15.82%,-6.01%)  "
```

Calculate mean counts/ 100,000 persons/per month for pre and post-announceent

```
age15_18$phase<-"pre_announcement"
age15_18$phase[which(age15_18$measurement_date>= "2016-03-01")]<-"post_announcement"

age15_18 %>% group_by (phase) %>%
  summarise (mean = mean (admission_prop, na.rm =T),
             (sd = sd (admission_prop, na.rm =T)))
```

```
# A tibble: 2 x 3
  phase              mean `(sd = sd(admission_prop, na.rm = T))`
  <chr>             <dbl>                                    <dbl>
1 post_announcement 11.3                                      1.99
2 pre_announcement   8.99                                     2.04
```

Asthma Analysis (IMD-5 - Least Deprived)

R code

- Code : Nina Rogers (Nina.Rogers@mrc-epid.cam.ac.uk)
- Code review : Chrissy h. Roberts (chrissy.roberts@LSHTM.ac.uk)

Data Prep and Functions Load

```
source("001_asthma_data_prep_age_sep.R")
source("007_asthma_deprivation_functions.R")
```

Filter the main Age-specific ITS dataset

```
least_deprived1_2012 <-  by_sep_asthma %>%
  filter(Social_deprivation_quintile5 == "Least Deprived 20%")
```

Chart admissions data

This provides a simple eyeball test of the data trend across time.

```
 ggplot(least_deprived1_2012, aes(x=measurement_date, y=admiss_quint_prop )) +
    geom_smooth() +
    xlab("")+
    ylab("Admissions per 100,000 population, per month")
```

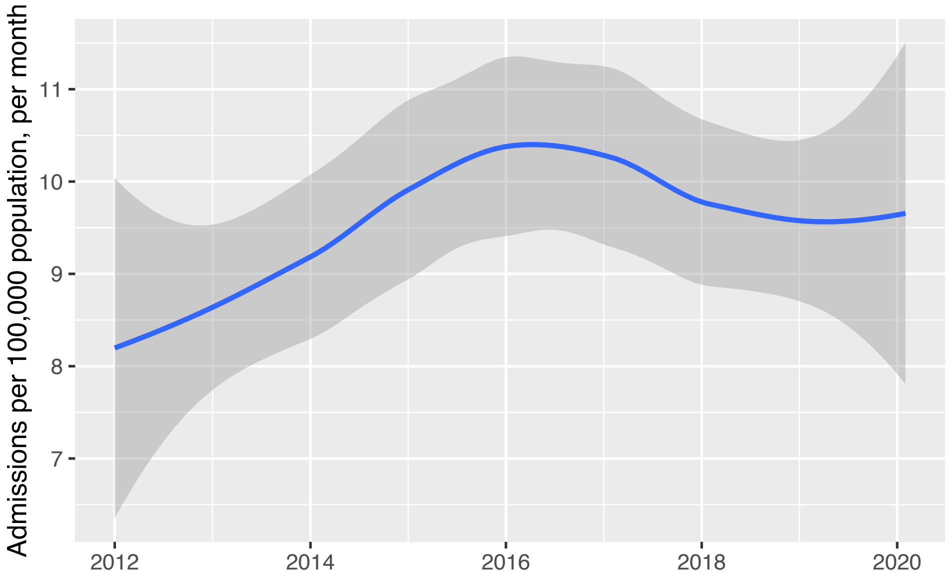

Create the time and intervention variables

This includes definition of time_months (study time), announcement.trend (change in trajectory post announcement) and imp.trend (change in trajectort post implementation).

```
least_deprived1_2012$time_months <-c(1:98)
least_deprived1_2012$announcement.trend <- c(rep(0,51),1:47)
least_deprived1_2012$imp.trend <- c(rep(0,75),1:23)
least_deprived1_2012 <- least_deprived1_2012 %>%
    select(measurement_date, time_months, everything())
```

Run OLS analysis

```
least_deprived1_2012.ols<-model_ols(df = least_deprived1_2012)
summary(least_deprived1_2012.ols)
```

```
Call:
lm(formula = admiss_quint_prop ~ time_months + announcement.trend +
    September + October + November + April + August + imp.trend,
    data = df)

Residuals:
    Min      1Q  Median      3Q     Max
-2.7783 -0.8638 -0.2406  0.6791  5.1423

Coefficients:
                   Estimate Std. Error t value Pr(>|t|)
(Intercept)         7.89672    0.39495  19.994  < 2e-16 ***
time_months         0.04937    0.01159   4.258 5.09e-05 ***
announcement.trend -0.09808    0.03144  -3.120 0.002438 **
September           4.51665    0.52741   8.564 3.00e-13 ***
October             1.70556    0.52759   3.233 0.001720 **
November            2.02557    0.52786   3.837 0.000232 ***
April              -2.11284    0.52788  -4.003 0.000129 ***
August             -4.10410    0.52732  -7.783 1.21e-11 ***
imp.trend           0.05435    0.05044   1.077 0.284206
---
Signif. codes:  0 '***' 0.001 '**' 0.01 '*' 0.05 '.' 0.1 ' ' 1

Residual standard error: 1.398 on 89 degrees of freedom
Multiple R-squared:  0.7115,    Adjusted R-squared:  0.6856
F-statistic: 27.44 on 8 and 89 DF,  p-value: < 2.2e-16
```

Run Durbin-Watson Test

```
least_deprived1_2012.dwt<-model_dwt(least_deprived1_2012.ols)
least_deprived1_2012.dwt
```

```
 lag Autocorrelation D-W Statistic p-value
   1    1.032395e-01      1.774078   0.192
   2    8.227048e-02      1.813735   0.268
   3    7.553624e-05      1.948420   0.774
   4   -2.920261e-02      2.003683   0.938
   5   -1.270651e-01      2.176012   0.312
   6   -2.311043e-01      2.379186   0.026
   7   -5.278440e-02      1.869722   0.808
   8   -6.138891e-02      1.875241   0.928
   9    3.504195e-02      1.670605   0.346
  10   -1.534487e-01      2.047270   0.330
  11    7.832821e-02      1.535900   0.144
  12    9.267036e-03      1.653013   0.216
 Alternative hypothesis: rho[lag] != 0
```

Chart acf of ols

```
graph.ols.acf.plots(model = least_deprived1_2012.ols)
```

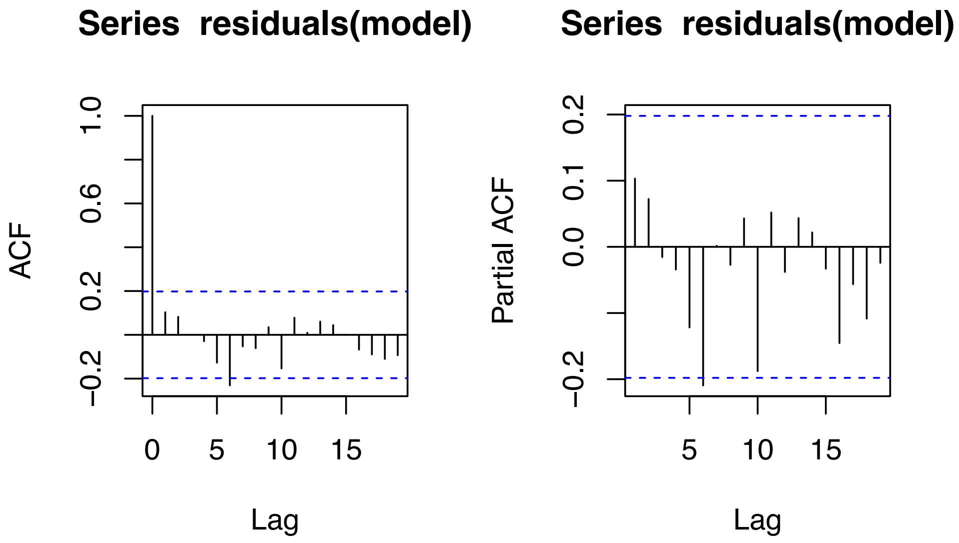

Run gls models with a variety of values of p and q

Run a variety of values here to provide belt and braces. Using values 1,2,3,4,6,8 and 12, allows for various cyclical calendar-relevant lags. fit.01 provides a linear relationship between x and y with no lag, whilst fit.02 provides a time-squared function without lag.

```
fit.01<-gls.modeller(df = least_deprived1_2012,timesquared = FALSE,
                     timecubed= FALSE,p=NULL,q=NULL)
fit.02<-gls.modeller(df = least_deprived1_2012,timesquared = TRUE,
                     timecubed= FALSE,p=NULL,q=NULL)

fit.03<-gls.modeller(df = least_deprived1_2012,timesquared = FALSE,
                     timecubed= FALSE,p=1,q=NULL)
fit.04<-gls.modeller(df = least_deprived1_2012,timesquared = FALSE,
                     timecubed= FALSE,p=2,q=NULL)
fit.05<-gls.modeller(df = least_deprived1_2012,timesquared = FALSE,
                     timecubed= FALSE,p=3,q=NULL)
fit.06<-gls.modeller(df = least_deprived1_2012,timesquared = FALSE,
                     timecubed= FALSE,p=4,q=NULL)
fit.07<-gls.modeller(df = least_deprived1_2012,timesquared = FALSE,
                     timecubed= FALSE,p=6,q=NULL)
fit.08<-gls.modeller(df = least_deprived1_2012,timesquared = FALSE,
                     timecubed= FALSE,p=8,q=NULL)
fit.09<-gls.modeller(df = least_deprived1_2012,timesquared = FALSE,
                     timecubed= FALSE,p=12,q=NULL)


fit.10<-gls.modeller(df = least_deprived1_2012,timesquared = FALSE,
                     timecubed= FALSE,p=NULL,q=1)
fit.11<-gls.modeller(df = least_deprived1_2012,timesquared = FALSE,
                     timecubed= FALSE,p=NULL,q=2)
fit.12<-gls.modeller(df = least_deprived1_2012,timesquared = FALSE,
                     timecubed= FALSE,p=NULL,q=3)
fit.13<-gls.modeller(df = least_deprived1_2012,timesquared = FALSE,
                     timecubed= FALSE,p=NULL,q=4)
fit.14<-gls.modeller(df = least_deprived1_2012,timesquared = FALSE,
                     timecubed= FALSE,p=NULL,q=6)
fit.15<-gls.modeller(df = least_deprived1_2012,timesquared = FALSE,
                     timecubed= FALSE,p=NULL,q=8)
fit.16<-gls.modeller(df = least_deprived1_2012,timesquared = FALSE,
                     timecubed= FALSE,p=NULL,q=12)
```

Compare the AIC values for various possible fits

```
models<-list(fit.01,fit.02,fit.03,fit.04,fit.05,fit.06,fit.07,fit.08,fit.09,fit.10,
             fit.11,fit.12,fit.13,fit.14,fit.15,fit.16)

least_deprived1_2012.aictab<-AICcmodavg::aictab(cand.set = models)
```

```
Warning in aictab.AICgls(cand.set = models):
Model names have been supplied automatically in the table

Warning in aictab.AICgls(cand.set = models):
Check model structure carefully as some models may be redundant
```

```
least_deprived1_2012.aictab
```

```
Model selection based on AICc:

       K   AICc Delta_AICc AICcWt Cum.Wt      LL
Mod14 16 353.00       0.00   0.64   0.64 -157.14
Mod1  10 356.86       3.86   0.09   0.73 -167.17
Mod2  10 356.86       3.86   0.09   0.83 -167.17
Mod3  11 358.24       5.24   0.05   0.87 -166.58
Mod10 11 358.41       5.41   0.04   0.91 -166.67
Mod15 18 358.90       5.89   0.03   0.95 -157.12
Mod11 12 360.15       7.15   0.02   0.97 -166.24
Mod4  12 360.23       7.23   0.02   0.98 -166.28
Mod5  13 362.78       9.78   0.00   0.99 -166.22
Mod12 13 362.80       9.80   0.00   0.99 -166.24
Mod7  16 364.28      11.28   0.00   1.00 -162.78
Mod16 22 364.61      11.61   0.00   1.00 -153.56
Mod6  14 365.06      12.06   0.00   1.00 -166.00
Mod13 14 365.50      12.50   0.00   1.00 -166.22
Mod8  18 369.86      16.86   0.00   1.00 -162.60
Mod9  22 374.63      21.63   0.00   1.00 -158.57
```

Fit.14 has the lowest AIC in this case, but has 16 components. Model 1 has 10 components and deltaAIC 3.86. There's likely to be a tradeoff in these metrics.

Keep the best model and delete the others

```
least_deprived1_2012.final.gls<-fit.01
rm(fit.01,fit.02,fit.03,fit.04,fit.05,fit.06,fit.07,fit.08,fit.09,fit.10,
   fit.11,fit.12,fit.13,fit.14,fit.15,fit.16,models)
```

Make table of results

```
least_deprived1_2012.gls.results.table<-gls.table.maker(least_deprived1_2012.final.gls)
least_deprived1_2012.gls.results.table
```

variable	Value	Std.Error	t-value	p-value
(Intercept)	7.89672	0.39495	19.99429	0.00000
time_months	0.04937	0.01159	4.25847	0.00005
announcement.trend	-0.09808	0.03144	-3.11996	0.00244
September	4.51665	0.52741	8.56378	0.00000
October	1.70556	0.52759	3.23271	0.00172
November	2.02557	0.52786	3.83731	0.00023
April	-2.11284	0.52788	-4.00251	0.00013
August	-4.10410	0.52732	-7.78287	0.00000
imp.trend	0.05435	0.05044	1.07742	0.28421

Get the fitted values from the model

```
least_deprived1_2012$predicted<-predict(least_deprived1_2012.final.gls)

tmp<-as.data.frame(predictSE.gls(mod = least_deprived1_2012.final.gls,
                                 newdata = least_deprived1_2012))
tmp$ci.min<-tmp$fit-(1.96*tmp$se.fit)
tmp$ci.max<-tmp$fit+(1.96*tmp$se.fit)
tmp<-select(tmp,ci.min,ci.max)

least_deprived1_2012$ci.min<-tmp$ci.min
least_deprived1_2012$ci.max<-tmp$ci.max
rm(tmp)
```

Add Counterfactuals 1 and 2 to the data table

```
least_deprived1_2012$cf<-NA

for(i in 1:nrow(least_deprived1_2012)){least_deprived1_2012$cf[i]<-
                                             counterfactual.function(i=i,
                                             df = least_deprived1_2012,
                                             model = least_deprived1_2012.final.gls)}

least_deprived1_2012$cf2<-NA

for(i in 1:nrow(least_deprived1_2012)){least_deprived1_2012$cf2[i]<-
                                             counterfactual2.function(i=i,
                                             df = least_deprived1_2012,
                                             model = least_deprived1_2012.final.gls)}
```

Chart the ITS result

```
least_deprived1_2012.its.chart<-its.model.plot(df = least_deprived1_2012,
                                    model = least_deprived1_2012.final.gls,
                                    points = FALSE,
                                    ylim.low = 0,
                                    ylim.high = 40,
                                    its.title="IMD 5 – Least Deprived",
                                    its.ytitle = "Incidence rates per 100,000 population"
least_deprived1_2012.its.chart
```

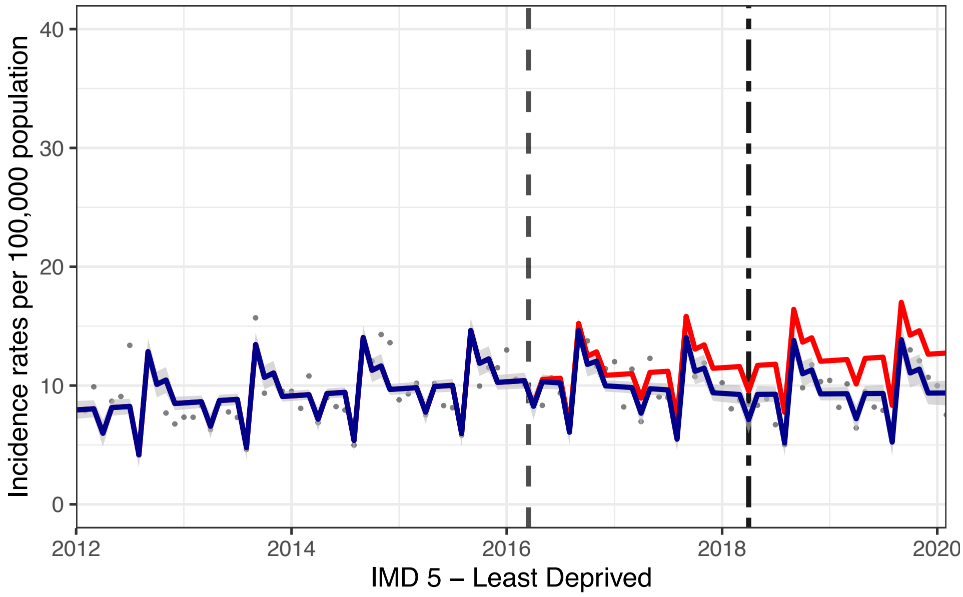

Get values of changes compared to counterfactual 1 (no intervention)

Note: The absolute difference takes no account for the confidence intervals on the counterfactual and is based only on the mean counterfactual values.

```
least_deprived1_2012.change.absolute <-get.predicted.anyweek(df = least_deprived1_2012,
                                                absrel = "abs",pos = 98)
least_deprived1_2012.change.absolute
```

```
[1] "-3.36, 95% CI -2.31,-4.41) | -26.38%, 95% CI -34.63%,-18.14%)  "
```

NB : Using Model 14 estimates these values at -3.96g (95% CI -3.24g,-4.69g) and -30.28% (95% CI -35.8%,-24.76%). Seems sensible to choose the model with the more conservative estimate, even if this is an underestimation of the true effect in this group.

Get values of changes compared to counterfactual 2 (no implementation)

Note: The absolute difference takes no account for the confidence intervals on the counterfactual and is based only on the mean counterfactual values.

```
least_deprived1_2012.change.absolute.cf2 <-
  get.predicted.anyweek.cf2(df = least_deprived1_2012,
  absrel = "abs",pos = 98)
least_deprived1_2012.change.absolute.cf2
```

```
[1] "1.25, 95% CI 2.3,0.2) | 15.39%, 95% CI 2.46%,28.31%)  "
```

Calculate mean counts/ 100,000 persons/per month for pre and post-announceent

```
least_deprived1_2012$phase<-"pre_announcement"
least_deprived1_2012$phase[which(least_deprived1_2012$measurement_date>= "2016-03-01")]<-
  "post_announcement"

least_deprived1_2012 %>% group_by (phase) %>%
  summarise (mean = mean (admiss_quint_prop, na.rm =T),
             (sd = sd (admiss_quint_prop, na.rm =T)))
```

```
# A tibble: 2 x 3
  phase               mean `(sd = sd(admiss_quint_prop, na.rm = T))`
  <chr>              <dbl>                                     <dbl>
1 post_announcement   9.78                                      2.38
2 pre_announcement    9.29                                      2.59
```

Asthma Analysis (IMD-4 - Less Deprived)

R code

- Code : Nina Rogers (Nina.Rogers@mrc-epid.cam.ac.uk)
- Code review : Chrissy h. Roberts (chrissy.roberts@LSHTM.ac.uk)

Data Prep and Functions Load

```
source("001_asthma_data_prep_age_sep.R")
source("007_asthma_deprivation_functions.R")
```

Filter the main Age-specific ITS dataset

```
less_deprived1_2012 <-  by_sep_asthma %>%
  filter(Social_deprivation_quintile5 == "Less Deprived 20%")
```

Chart admissions data

This provides a simple eyeball test of the data trend across time.

```
ggplot(less_deprived1_2012, aes(x=measurement_date, y=admiss_quint_prop )) +
   geom_smooth() +
   xlab("")+
   ylab("Admissions per 100,000 population, per month")
```

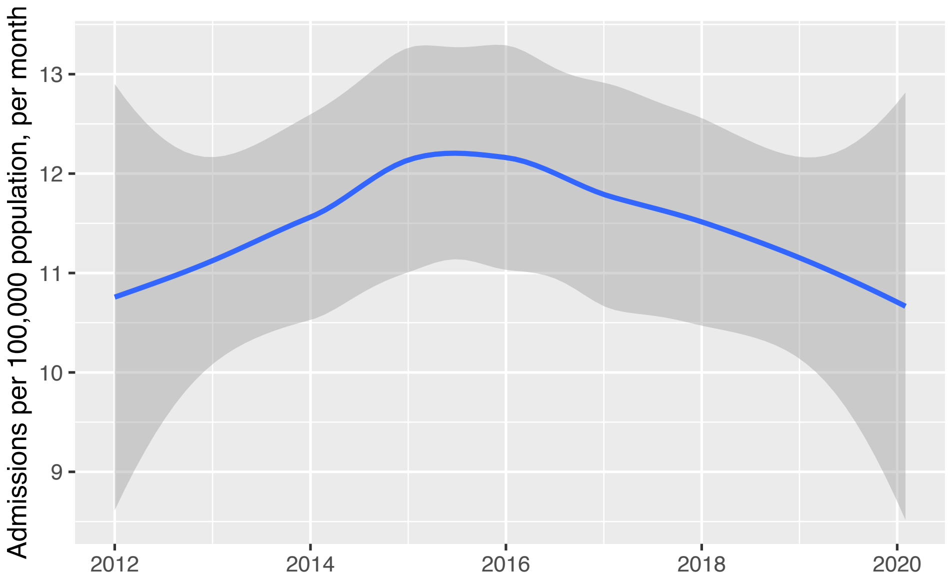

Create the time and intervention variables

This includes definition of time_months (study time), announcement.trend (change in trajectory post announcement) and imp.trend (change in trajectort post implementation).

```
less_deprived1_2012$time_months <-c(1:98)
less_deprived1_2012$announcement.trend <- c(rep(0,51),1:47)
less_deprived1_2012$imp.trend <- c(rep(0,75),1:23)
less_deprived1_2012 <- less_deprived1_2012 %>%
  select(measurement_date, time_months, everything())
```

Run OLS analysis

```
less_deprived1_2012.ols<-model_ols(df = less_deprived1_2012)
summary(less_deprived1_2012.ols)
```

```
Call:
lm(formula = admiss_quint_prop ~ time_months + announcement.trend +
    September + October + November + April + August + imp.trend,
    data = df)

Residuals:
    Min      1Q  Median      3Q     Max
-3.8509 -1.0545 -0.1209  0.7349  4.9574

Coefficients:
                   Estimate Std. Error t value Pr(>|t|)
(Intercept)        10.72040    0.42658  25.131  < 2e-16 ***
time_months         0.02528    0.01252   2.019  0.04652 *
announcement.trend -0.05404    0.03395  -1.592  0.11503
September           4.65655    0.56965   8.174 1.90e-12 ***
October             2.28058    0.56985   4.002  0.00013 ***
November            3.27510    0.57014   5.744 1.27e-07 ***
April              -2.75365    0.57015  -4.830 5.65e-06 ***
August             -5.02854    0.56956  -8.829 8.49e-14 ***
imp.trend          -0.02094    0.05448  -0.384  0.70158
---
Signif. codes:  0 '***' 0.001 '**' 0.01 '*' 0.05 '.' 0.1 ' ' 1

Residual standard error: 1.51 on 89 degrees of freedom
Multiple R-squared:  0.7404,    Adjusted R-squared:  0.717
F-statistic: 31.72 on 8 and 89 DF,  p-value: < 2.2e-16
```

Run Durbin-Watson Test

```
less_deprived1_2012.dwt<-model_dwt(less_deprived1_2012.ols)
less_deprived1_2012.dwt
```

```
 lag Autocorrelation D-W Statistic p-value
   1     0.165377882      1.644860   0.048
   2     0.039320923      1.893388   0.468
   3     0.045364973      1.878827   0.560
   4    -0.065355301      2.093371   0.632
   5    -0.101723302      2.161913   0.378
   6    -0.228437749      2.414011   0.014
   7    -0.131963229      2.099813   0.368
   8    -0.116533013      2.050152   0.428
   9     0.042566694      1.728741   0.520
  10    -0.027848115      1.866647   0.954
  11    -0.006873371      1.780700   0.748
  12     0.097550575      1.555550   0.094
 Alternative hypothesis: rho[lag] != 0
```

Chart acf of ols

```
graph.ols.acf.plots(model = less_deprived1_2012.ols)
```

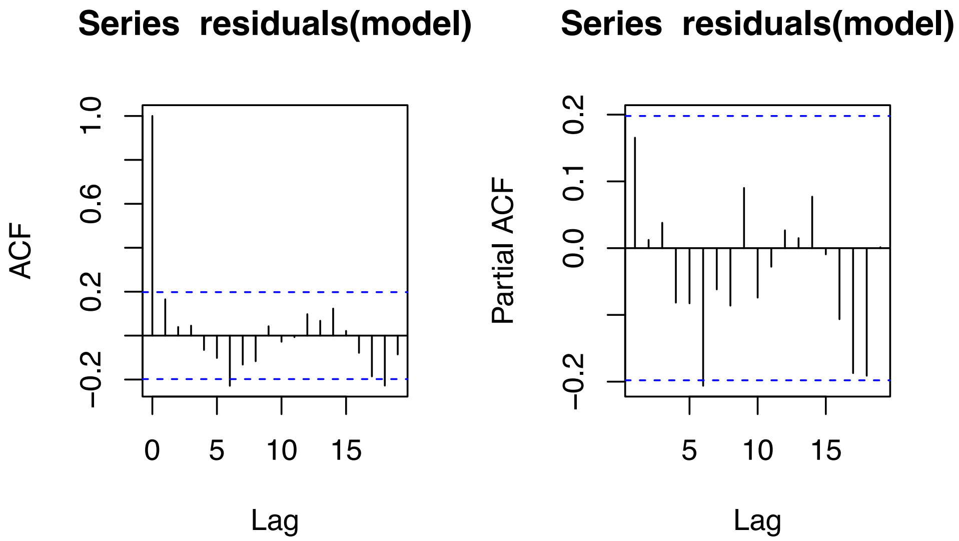

Run gls models with a variety of values of p and q

Run a variety of values here to provide belt and braces. Using values 1,2,3,4,6,8 and 12, allows for various cyclical calendar-relevant lags. fit.01 provides a linear relationship between x and y with no lag, whilst fit.02 provides a time-squared function without lag.

```
fit.01<-gls.modeller(df = less_deprived1_2012,
                    timesquared = FALSE,timecubed = FALSE,p=NULL,q=NULL)
fit.02<-gls.modeller(df = less_deprived1_2012,
                    timesquared = TRUE,timecubed = FALSE,p=NULL,q=NULL)
fit.03<-gls.modeller(df = less_deprived1_2012,
                    timesquared = FALSE,timecubed = FALSE,p=1,q=NULL)
fit.04<-gls.modeller(df = less_deprived1_2012,
                    timesquared = FALSE,timecubed = FALSE,p=2,q=NULL)
fit.05<-gls.modeller(df = less_deprived1_2012,
                    timesquared = FALSE,timecubed = FALSE,p=3,q=NULL)
fit.06<-gls.modeller(df = less_deprived1_2012,
                    timesquared = FALSE,timecubed = FALSE,p=4,q=NULL)
fit.07<-gls.modeller(df = less_deprived1_2012,
                    timesquared = FALSE,timecubed = FALSE,p=6,q=NULL)
fit.08<-gls.modeller(df = less_deprived1_2012,
                    timesquared = FALSE,timecubed = FALSE,p=8,q=NULL)
fit.09<-gls.modeller(df = less_deprived1_2012,
                    timesquared = FALSE,timecubed = FALSE,p=12,q=NULL)
fit.10<-gls.modeller(df = less_deprived1_2012,
                    timesquared = FALSE,timecubed = FALSE,p=NULL,q=1)
fit.11<-gls.modeller(df = less_deprived1_2012,
                    timesquared = FALSE,timecubed = FALSE,p=NULL,q=2)
fit.12<-gls.modeller(df = less_deprived1_2012,
                    timesquared = FALSE,timecubed = FALSE,p=NULL,q=3)
fit.13<-gls.modeller(df = less_deprived1_2012,
                    timesquared = FALSE,timecubed = FALSE,p=NULL,q=4)
fit.14<-gls.modeller(df = less_deprived1_2012,
                    timesquared = FALSE,timecubed = FALSE,p=NULL,q=6)
fit.15<-gls.modeller(df = less_deprived1_2012,
                    timesquared = FALSE,timecubed = FALSE,p=NULL,q=8)
#fit.16<-gls.modeller(df = less_deprived1_2012,
# timesquared = FALSE,timecubed = FALSE,p = NULL,q=12)
```

Model 16 did not converge

Compare the AIC values for various possible fits

```
models<-list(fit.01,fit.02,fit.03,fit.04,fit.05,fit.06,fit.07,fit.08,fit.09,fit.10,
             fit.11,fit.12,fit.13,fit.14,fit.15)

less_deprived1_2012.aictab<-AICcmodavg::aictab(cand.set = models)
```

```
Warning in aictab.AICgls(cand.set = models):
Model names have been supplied automatically in the table

Warning in aictab.AICgls(cand.set = models):
Check model structure carefully as some models may be redundant
```

```
less_deprived1_2012.aictab
```

```
Model selection based on AICc:

       K   AICc Delta_AICc AICcWt Cum.Wt      LL
Mod3  11 371.46       0.00   0.20   0.20 -173.19
Mod10 11 371.54       0.09   0.19   0.39 -173.24
Mod1  10 371.96       0.50   0.16   0.55 -174.72
Mod2  10 371.96       0.50   0.16   0.71 -174.72
Mod14 16 372.50       1.04   0.12   0.83 -166.89
Mod4  12 374.05       2.59   0.06   0.88 -173.19
Mod11 12 374.11       2.65   0.05   0.93 -173.22
Mod12 13 376.18       4.72   0.02   0.95 -172.92
Mod5  13 376.62       5.16   0.02   0.97 -173.14
Mod15 18 377.04       5.58   0.01   0.98 -166.19
Mod6  14 378.31       6.85   0.01   0.99 -172.62
Mod13 14 378.59       7.13   0.01   0.99 -172.76
Mod7  16 378.87       7.41   0.00   1.00 -170.08
Mod8  18 380.20       8.74   0.00   1.00 -167.77
Mod9  22 390.25      18.79   0.00   1.00 -166.38
```

Keep the best model and delete the others

```
less_deprived1_2012.final.gls<-fit.03
rm(fit.01,fit.02,fit.03,fit.04,fit.05,fit.06,fit.07,fit.08,fit.09,fit.10,
   fit.11,fit.12,fit.13,fit.14,fit.15,models)
```

Make table of results

```
less_deprived1_2012.gls.results.table<-gls.table.maker(less_deprived1_2012.final.gls)
less_deprived1_2012.gls.results.table
```

variable	Value	Std.Error	t-value	p-value
(Intercept)	10.76221	0.50538	21.29525	0.00000
time_months	0.02480	0.01501	1.65272	0.10191
announcement.trend	-0.05240	0.04078	-1.28493	0.20215
September	4.65056	0.57705	8.05916	0.00000
October	2.22462	0.57727	3.85371	0.00022
November	3.04757	0.55745	5.46701	0.00000
April	-2.88282	0.53734	-5.36494	0.00000
August	-4.92299	0.55699	-8.83857	0.00000
imp.trend	-0.02572	0.06508	-0.39514	0.69369

Get the fitted values from the model

```
less_deprived1_2012$predicted<-predict(less_deprived1_2012.final.gls)

tmp<-as.data.frame(predictSE.gls(mod = less_deprived1_2012.final.gls,
                                 newdata = less_deprived1_2012))
tmp$ci.min<-tmp$fit-(1.96*tmp$se.fit)
tmp$ci.max<-tmp$fit+(1.96*tmp$se.fit)
tmp<-select(tmp,ci.min,ci.max)

less_deprived1_2012$ci.min<-tmp$ci.min
less_deprived1_2012$ci.max<-tmp$ci.max
rm(tmp)
```

Add Counterfactuals 1 and 2 to the data table

```
less_deprived1_2012$cf<-NA

for(i in 1:nrow(less_deprived1_2012)){less_deprived1_2012$cf[i]<-
  counterfactual.function(i=i,
  df = less_deprived1_2012,
  model = less_deprived1_2012.final.gls)}

less_deprived1_2012$cf2<-NA

for(i in 1:nrow(less_deprived1_2012)){less_deprived1_2012$cf2[i]<-
  counterfactual2.function(i=i,
  df = less_deprived1_2012,
  model = less_deprived1_2012.final.gls)}
```

Chart the ITS result

```
less_deprived1_2012.its.chart<-its.model.plot(df = less_deprived1_2012,model =
less_deprived1_2012.final.gls,
  ylim.low = 0,ylim.high = 40,
  its.title="IMD 4 - Less Deprived",
  its.ytitle = "Incidence rates per 100,000 population" )
less_deprived1_2012.its.chart
```

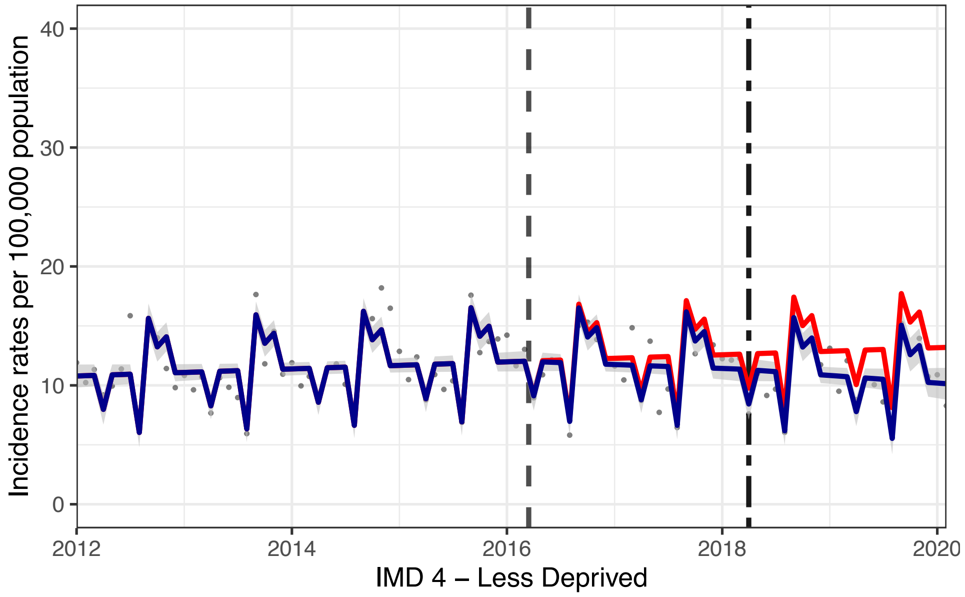

Get values of changes compared to counterfactual 1 (no intervention)

Note: The absolute difference takes no account for the confidence intervals on the counterfactual and is based only on the mean counterfactual values.

```
less_deprived1_2012.change.absolute <-
  get.predicted.anyweek(df = less_deprived1_2012,
    absrel = "abs",pos = 98)

less_deprived1_2012.change.absolute
```

```
[1] "-3.05, 95% CI -1.72,-4.39) | -23.15%, 95% CI -33.29%,-13.01%)  "
```

Get values of changes compared to counterfactual 2 (no implementation)

Note: The absolute difference takes no account for the confidence intervals on the counterfactual and is based only on the mean counterfactual values.

```
less_deprived1_2012.change.absolute.cf2 <-
  get.predicted.anyweek.cf2(df = less_deprived1_2012,
    absrel = "abs",pos = 98)
less_deprived1_2012.change.absolute.cf2
```

```
[1] "-0.59, 95% CI 0.75,-1.93) | -5.51%, 95% CI -17.98%,6.96%)  "
```

Calculate mean counts/ 100,000 persons/per month for pre and post-announceent

```
less_deprived1_2012$phase<-"pre_announcement"
less_deprived1_2012$phase[which(less_deprived1_2012$measurement_date>= "2016-03-01")]<-
  "post_announcement"

less_deprived1_2012 %>% group_by (phase) %>%
  summarise (mean = mean (admiss_quint_prop, na.rm =T),
             (sd = sd (admiss_quint_prop, na.rm =T))
             )
```

```
# A tibble: 2 x 3
  phase              mean `(sd = sd(admiss_quint_prop, na.rm = T))`
  <chr>             <dbl>                                     <dbl>
1 post_announcement  11.4                                      2.79
2 pre_announcement   11.6                                      2.91
```

Asthma Analysis (IMD-3 - Mid Deprived)

R code

- Code : Nina Rogers (Nina.Rogers@mrc-epid.cam.ac.uk)
- Code review : Chrissy h. Roberts (chrissy.roberts@LSHTM.ac.uk)

Data Prep and Functions Load

```
source("001_asthma_data_prep_age_sep.R")
source("007_asthma_deprivation_functions.R")
```

Filter the main Age-specific ITS dataset

```
mid_deprived1_2012 <-  by_sep_asthma %>%
  filter(Social_deprivation_quintile5 == "Mid Deprived 20%")
```

Chart admissions data

This provides a simple eyeball test of the data trend across time.

```
 ggplot(mid_deprived1_2012, aes(x=measurement_date, y=admiss_quint_prop )) +
    geom_smooth() +
    xlab("")+
    ylab("Admissions per 100,000 population, per month")
```

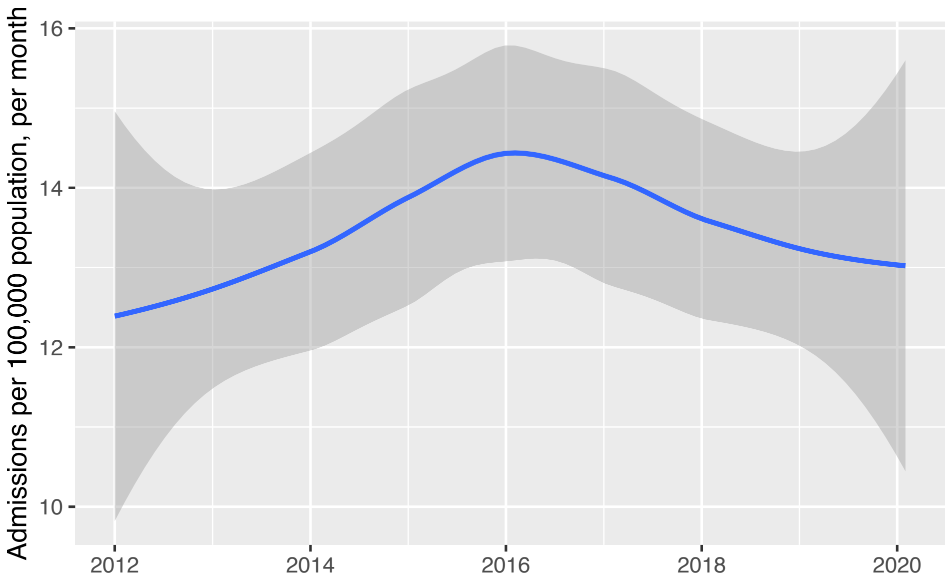

Create the time and intervention variables

This includes definition of time_months (study time), announcement.trend (change in trajectory post announcement) and imp.trend (change in trajectort post implementation).

```
mid_deprived1_2012$time_months <-c(1:98)
mid_deprived1_2012$announcement.trend <- c(rep(0,51),1:47)
mid_deprived1_2012$imp.trend <- c(rep(0,75),1:23)
mid_deprived1_2012 <- mid_deprived1_2012 %>%
  select(measurement_date, time_months, everything())
```

Run OLS analysis

```
mid_deprived1_2012.ols<-model_ols(df = mid_deprived1_2012)
summary(mid_deprived1_2012.ols)
```

```
Call:
lm(formula = admiss_quint_prop ~ time_months + announcement.trend +
    September + October + November + April + August + imp.trend,
    data = df)

Residuals:
    Min      1Q  Median      3Q     Max
-2.9989 -1.3929 -0.2593  0.9969  6.7929

Coefficients:
                   Estimate Std. Error t value Pr(>|t|)
(Intercept)        12.03225    0.52737  22.815  < 2e-16 ***
time_months         0.04342    0.01548   2.805 0.006178 **
announcement.trend -0.09085    0.04198  -2.164 0.033118 *
September           5.69135    0.70425   8.081 2.96e-12 ***
October             3.09270    0.70449   4.390 3.11e-05 ***
November            3.22213    0.70485   4.571 1.56e-05 ***
April              -2.76961    0.70487  -3.929 0.000168 ***
August             -6.21057    0.70414  -8.820 8.85e-14 ***
imp.trend           0.02147    0.06736   0.319 0.750625
---
Signif. codes:  0 '***' 0.001 '**' 0.01 '*' 0.05 '.' 0.1 ' ' 1

Residual standard error: 1.867 on 89 degrees of freedom
Multiple R-squared:  0.7252,    Adjusted R-squared:  0.7005
F-statistic: 29.36 on 8 and 89 DF,  p-value: < 2.2e-16
```

Run Durbin-Watson Test

```
mid_deprived1_2012.dwt<-model_dwt(mid_deprived1_2012.ols)
mid_deprived1_2012.dwt
```

```
 lag Autocorrelation D-W Statistic p-value
   1     0.195045669      1.607746   0.024
   2     0.011648726      1.963092   0.748
   3     0.009911269      1.957317   0.832
   4    -0.033752898      2.025908   0.832
   5    -0.165287573      2.286522   0.104
   6    -0.176006880      2.272529   0.092
   7    -0.114040951      1.998899   0.744
   8    -0.116711836      1.970784   0.748
   9    -0.036312236      1.790192   0.692
  10    -0.031900225      1.755018   0.594
  11     0.045352531      1.583324   0.268
  12    -0.008728924      1.660324   0.228
 Alternative hypothesis: rho[lag] != 0
```

Chart acf of ols

```
graph.ols.acf.plots(model = mid_deprived1_2012.ols)
```

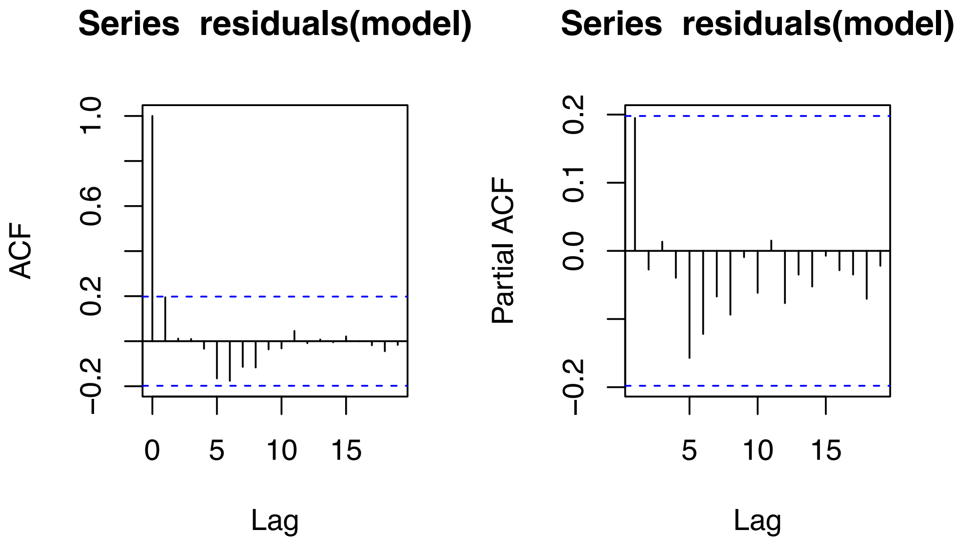

Run gls models with a variety of values of p and q

Run a variety of values here to provide belt and braces. Using values 1,2,3,4,6,8 and 12, allows for various cyclical calendar-relevant lags. fit.01 provides a linear relationship between x and y with no lag, whilst fit.02 provides a time-squared function without lag.

```
fit.01<-gls.modeller(df = mid_deprived1_2012,
                     timesquared= FALSE,timecubed= FALSE,p=NULL,q=NULL)
fit.02<-gls.modeller(df = mid_deprived1_2012,
                     timesquared= TRUE,timecubed= FALSE,p=NULL,q=NULL)

fit.03<-gls.modeller(df = mid_deprived1_2012,
                     timesquared= FALSE,timecubed= FALSE,p=1,q=NULL)
fit.04<-gls.modeller(df = mid_deprived1_2012,
                     timesquared= FALSE,timecubed= FALSE,p=2,q=NULL)
fit.05<-gls.modeller(df = mid_deprived1_2012,
                     timesquared= FALSE,timecubed= FALSE,p=3,q=NULL)
fit.06<-gls.modeller(df = mid_deprived1_2012,
                     timesquared= FALSE,timecubed= FALSE,p=4,q=NULL)
fit.07<-gls.modeller(df = mid_deprived1_2012,
                     timesquared= FALSE,timecubed= FALSE,p=6,q=NULL)
fit.08<-gls.modeller(df = mid_deprived1_2012,
                     timesquared= FALSE,timecubed= FALSE,p=8,q=NULL)
fit.09<-gls.modeller(df = mid_deprived1_2012,
                     timesquared= FALSE,timecubed= FALSE,p=12,q=NULL)
fit.10<-gls.modeller(df = mid_deprived1_2012,
                     timesquared= FALSE,timecubed= FALSE,p=NULL,q=1)
fit.11<-gls.modeller(df = mid_deprived1_2012,
                     timesquared= FALSE,timecubed= FALSE,p=NULL,q=2)
fit.12<-gls.modeller(df = mid_deprived1_2012,
                     timesquared= FALSE,timecubed= FALSE,p=NULL,q=3)
fit.13<-gls.modeller(df = mid_deprived1_2012,
                     timesquared= FALSE,timecubed= FALSE,p=NULL,q=4)
fit.14<-gls.modeller(df = mid_deprived1_2012,
                     timesquared= FALSE,timecubed= FALSE,p=NULL,q=6)
fit.15<-gls.modeller(df = mid_deprived1_2012,
                     timesquared= FALSE,timecubed= FALSE,p=NULL,q=8)
fit.16<-gls.modeller(df = mid_deprived1_2012,
                     timesquared= FALSE,timecubed= FALSE,p=NULL,q=12)
```

Compare the AIC values for various possible fits

```
models<-list(fit.01,fit.02,fit.03,fit.04,fit.05,fit.06,fit.07,fit.08,fit.09,fit.10,
             fit.11,fit.12,fit.13,fit.14,fit.15,fit.16)

mid_deprived1_2012.aictab<-AICcmodavg::aictab(cand.set = models)
```

```
Warning in aictab.AICgls(cand.set = models):
Model names have been supplied automatically in the table
```

```
Warning in aictab.AICgls(cand.set = models):
Check model structure carefully as some models may be redundant
```

```
mid_deprived1_2012.aictab
```

```
Model selection based on AICc:

       K   AICc Delta_AICc AICcWt Cum.Wt      LL
Mod10 11 411.48       0.00   0.27   0.27 -193.20
Mod3  11 411.79       0.31   0.23   0.49 -193.36
Mod1  10 413.53       2.06   0.09   0.59 -195.50
Mod2  10 413.53       2.06   0.09   0.68 -195.50
Mod4  12 414.05       2.57   0.07   0.75 -193.19
Mod11 12 414.08       2.60   0.07   0.83 -193.20
Mod14 16 414.20       2.73   0.07   0.89 -187.74
Mod15 18 415.08       3.60   0.04   0.94 -185.21
Mod12 13 416.62       5.15   0.02   0.96 -193.14
Mod5  13 416.71       5.23   0.02   0.98 -193.19
Mod16 22 418.66       7.19   0.01   0.99 -180.59
Mod13 14 419.05       7.57   0.01   0.99 -192.99
Mod6  14 419.37       7.89   0.01   1.00 -193.16
Mod7  16 420.50       9.02   0.00   1.00 -190.89
Mod8  18 424.55      13.07   0.00   1.00 -189.95
Mod9  22 432.27      20.80   0.00   1.00 -187.39
```

Keep the best model and delete the others

```
mid_deprived1_2012.final.gls<-fit.10
rm(fit.01,fit.02,fit.03,fit.04,fit.05,fit.06,fit.07,fit.08,fit.09,fit.10,
   fit.11,fit.12,fit.13,fit.14,fit.15,fit.16,models)
```

Make table of results

```
mid_deprived1_2012.gls.results.table<-gls.table.maker(mid_deprived1_2012.final.gls)
mid_deprived1_2012.gls.results.table
```

variable	Value	Std.Error	t-value	p-value
(Intercept)	12.10031	0.62661	19.31089	0.00000
time_months	0.04378	0.01860	2.35307	0.02082
announcement.trend	-0.09129	0.05055	-1.80609	0.07429
September	5.61137	0.71987	7.79497	0.00000
October	3.01267	0.72020	4.18310	0.00007
November	2.84083	0.68398	4.15336	0.00007
April	-3.25264	0.64979	-5.00566	0.00000
August	-6.17184	0.68320	-9.03373	0.00000
imp.trend	0.02115	0.08073	0.26197	0.79395

Get the fitted values from the model

```
mid_deprived1_2012$predicted<-predict(mid_deprived1_2012.final.gls)

tmp<-as.data.frame(predictSE.gls(mod = mid_deprived1_2012.final.gls,
                                  newdata = mid_deprived1_2012))
tmp$ci.min<-tmp$fit-(1.96*tmp$se.fit)
tmp$ci.max<-tmp$fit+(1.96*tmp$se.fit)
tmp<-select(tmp,ci.min,ci.max)

mid_deprived1_2012$ci.min<-tmp$ci.min
mid_deprived1_2012$ci.max<-tmp$ci.max
rm(tmp)
```

Add Counterfactuals 1 and 2 to the data table

```
mid_deprived1_2012$cf<-NA

for(i in 1:nrow(mid_deprived1_2012)){mid_deprived1_2012$cf[i]<-
  counterfactual.function(i=i,
    df = mid_deprived1_2012,
    model = mid_deprived1_2012.final.gls)}

mid_deprived1_2012$cf2<-NA

for(i in 1:nrow(mid_deprived1_2012)){mid_deprived1_2012$cf2[i]<-
  counterfactual2.function(i=i,
    df = mid_deprived1_2012,
    model = mid_deprived1_2012.final.gls)}
```

Chart the ITS result

```
mid_deprived1_2012.its.chart<-its.model.plot(
  df = mid_deprived1_2012,
  model = mid_deprived1_2012.final.gls,points = FALSE,
  ylim.low = 0,
  ylim.high = 40,
  its.title="IMD 3 – Mid-level Deprivation",
  its.ytitle = "Incidence rates per 100,000 population" )
mid_deprived1_2012.its.chart
```

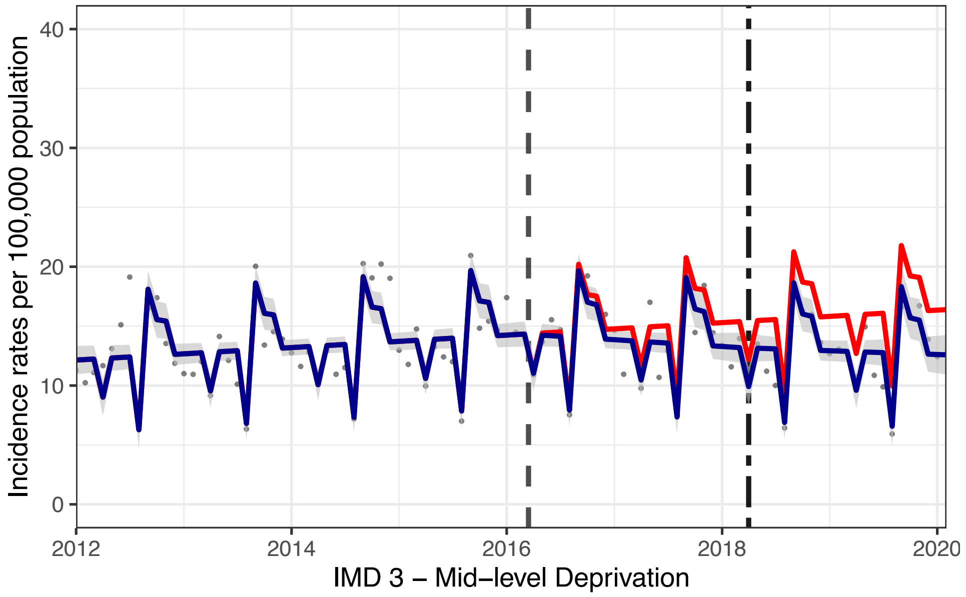

Get values of changes compared to counterfactual 1 (no intervention)

Note: The absolute difference takes no account for the confidence intervals on the counterfactual and is based only on the mean counterfactual values.

```
mid_deprived1_2012.change.absolute <-get.predicted.anyweek(
  df = mid_deprived1_2012,
  absrel = "abs",pos = 98)
mid_deprived1_2012.change.absolute
```

```
[1] "-3.8, 95% CI -2.14,-5.47) | -23.21%, 95% CI -33.35%,-13.07%)  "
```

Get values of changes compared to counterfactual 2 (no implementation)

Note: The absolute difference takes no account for the confidence intervals on the counterfactual and is based only on the mean counterfactual values.

```
mid_deprived1_2012.change.absolute.cf2 <-get.predicted.anyweek.cf2(
  df = mid_deprived1_2012,
  absrel = "abs",pos = 98)
mid_deprived1_2012.change.absolute.cf2
```

```
[1] "0.49, 95% CI 2.15,-1.18) | 4.02%, 95% CI -9.72%,17.76%)  "
```

Calculate mean counts/ 100,000 persons/per month for pre and post-announceent

```
mid_deprived1_2012$phase<-"pre_announcement"
mid_deprived1_2012$phase[which(mid_deprived1_2012$measurement_date>= "2016-03-01")]<-
  "post_announcement"

mid_deprived1_2012 %>% group_by (phase) %>%
  summarise (mean = mean (admiss_quint_prop, na.rm =T),
             (sd = sd (admiss_quint_prop, na.rm =T)))
```

```
# A tibble: 2 x 3
  phase              mean `(sd = sd(admiss_quint_prop, na.rm = T))`
  <chr>             <dbl>                                     <dbl>
1 post_announcement  13.6                                      3.29
2 pre_announcement   13.3                                      3.56
```

Asthma Analysis (IMD-2 - More Deprived)

R code

- Code : Nina Rogers (Nina.Rogers@mrc-epid.cam.ac.uk)
- Code review : Chrissy h. Roberts (chrissy.roberts@LSHTM.ac.uk)

Data Prep and Functions Load

```
source("001_asthma_data_prep_age_sep.R")
source("007_asthma_deprivation_functions.R")
```

Filter the main Age-specific ITS dataset

```
more_deprived1_2012 <-  by_sep_asthma %>%
  filter(Social_deprivation_quintile5 == "More Deprived 20%")
```

Chart admissions data

This provides a simple eyeball test of the data trend across time.

```
 ggplot(more_deprived1_2012, aes(x=measurement_date, y=admiss_quint_prop )) +
    geom_smooth() +
    xlab("")+
    ylab("Admissions per 100,000 population, per month")
```

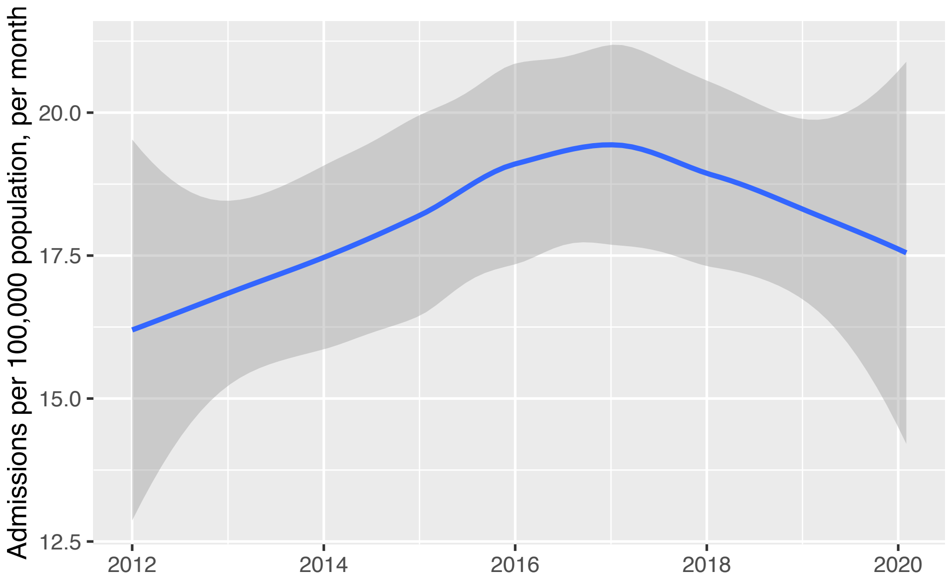

Create the time and intervention variables

This includes definition of time_months (study time), announcement.trend (change in trajectory post announcement) and imp.trend (change in trajectort post implementation).

```
more_deprived1_2012$time_months <-c(1:98)
more_deprived1_2012$announcement.trend <- c(rep(0,51),1:47)
more_deprived1_2012$imp.trend <- c(rep(0,75),1:23)
more_deprived1_2012 <- more_deprived1_2012 %>%
  select(measurement_date, time_months, everything())
```

Run OLS analysis

```
more_deprived1_2012.ols<-model_ols(df = more_deprived1_2012)
summary(more_deprived1_2012.ols)
```

```
Call:
lm(formula = admiss_quint_prop ~ time_months + announcement.trend +
    September + October + November + April + August + imp.trend,
    data = df)

Residuals:
    Min      1Q  Median      3Q     Max
-4.7810 -1.4472 -0.0391  1.2488  8.3785

Coefficients:
                   Estimate Std. Error t value Pr(>|t|)
(Intercept)        15.89564    0.64306  24.719  < 2e-16 ***
time_months         0.06101    0.01887   3.232  0.00172 **
announcement.trend -0.08709    0.05119  -1.702  0.09234 .
September           7.73905    0.85875   9.012 3.55e-14 ***
October             3.81100    0.85904   4.436 2.61e-05 ***
November            3.97894    0.85948   4.629 1.24e-05 ***
April              -3.75024    0.85950  -4.363 3.44e-05 ***
August             -8.28548    0.85860  -9.650 1.69e-15 ***
imp.trend          -0.03029    0.08213  -0.369  0.71317
---
Signif. codes:  0 '***' 0.001 '**' 0.01 '*' 0.05 '.' 0.1 ' ' 1

Residual standard error: 2.276 on 89 degrees of freedom
Multiple R-squared:  0.7591,    Adjusted R-squared:  0.7375
F-statistic: 35.07 on 8 and 89 DF,  p-value: < 2.2e-16
```

Run Durbin-Watson Test

```
more_deprived1_2012.dwt<-model_dwt(more_deprived1_2012.ols)
more_deprived1_2012.dwt
```

```
 lag Autocorrelation D-W Statistic p-value
   1      0.21014815      1.577398   0.010
   2     -0.01605103      2.021031   0.910
   3      0.06300789      1.857667   0.474
   4     -0.01579571      2.010553   0.902
   5     -0.06701130      2.112645   0.528
   6     -0.12838326      2.227738   0.136
   7      0.04421485      1.730086   0.350
   8     -0.10287457      1.982298   0.688
   9     -0.15765883      2.084077   0.300
  10     -0.10150949      1.951506   0.606
  11     -0.12566012      1.978453   0.474
  12      0.03008683      1.632284   0.206
 Alternative hypothesis: rho[lag] != 0
```

Chart acf of ols

```
graph.ols.acf.plots(model = more_deprived1_2012.ols)
```

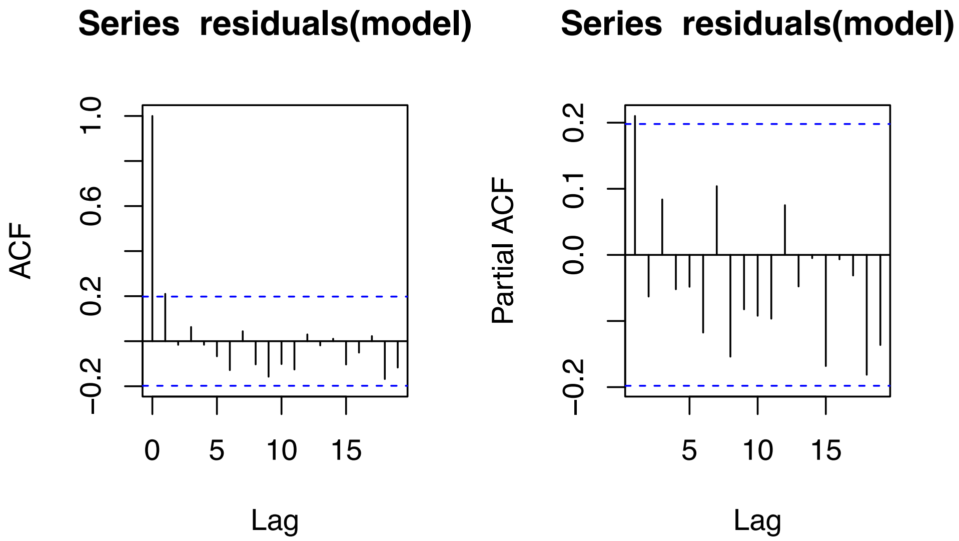

Run gls models with a variety of values of p and q

Run a variety of values here to provide belt and braces. Using values 1,2,3,4,6,8 and 12, allows for various cyclical calendar-relevant lags. fit.01 provides a linear relationship between x and y with no lag, whilst fit.02 provides a time-squared function without lag.

```
fit.01<-gls.modeller(df = more_deprived1_2012,
                     timesquared= FALSE,timecubed= FALSE,p=NULL,q=NULL)
fit.02<-gls.modeller(df = more_deprived1_2012,
                     timesquared= TRUE,timecubed= FALSE,p=NULL,q=NULL)

fit.03<-gls.modeller(df = more_deprived1_2012,
                     timesquared= FALSE,timecubed= FALSE,p=1,q=NULL)
fit.04<-gls.modeller(df = more_deprived1_2012,
                     timesquared= FALSE,timecubed= FALSE,p=2,q=NULL)
fit.05<-gls.modeller(df = more_deprived1_2012,
                     timesquared= FALSE,timecubed= FALSE,p=3,q=NULL)
fit.06<-gls.modeller(df = more_deprived1_2012,
                     timesquared= FALSE,timecubed= FALSE,p=4,q=NULL)
fit.07<-gls.modeller(df = more_deprived1_2012,
                     timesquared= FALSE,timecubed= FALSE,p=6,q=NULL)
fit.08<-gls.modeller(df = more_deprived1_2012,
                     timesquared= FALSE,timecubed= FALSE,p=8,q=NULL)
fit.09<-gls.modeller(df = more_deprived1_2012,
                     timesquared= FALSE,timecubed= FALSE,p=12,q=NULL)
fit.10<-gls.modeller(df = more_deprived1_2012,
                     timesquared= FALSE,timecubed= FALSE,p=NULL,q=1)
fit.11<-gls.modeller(df = more_deprived1_2012,
                     timesquared= FALSE,timecubed= FALSE,p=NULL,q=2)
fit.12<-gls.modeller(df = more_deprived1_2012,
                     timesquared= FALSE,timecubed= FALSE,p=NULL,q=3)
fit.13<-gls.modeller(df = more_deprived1_2012,
                     timesquared= FALSE,timecubed= FALSE,p=NULL,q=4)
fit.14<-gls.modeller(df = more_deprived1_2012,
                     timesquared= FALSE,timecubed= FALSE,p=NULL,q=6)
fit.15<-gls.modeller(df = more_deprived1_2012,
                     timesquared= FALSE,timecubed= FALSE,p=NULL,q=8)
fit.16<-gls.modeller(df = more_deprived1_2012,
                     timesquared= FALSE,timecubed= FALSE,p=NULL,q=12)
```

Compare the AIC values for various possible fits

```
models<-list(fit.01,fit.02,fit.03,fit.04,fit.05,fit.06,fit.07,fit.08,fit.09,fit.10,
             fit.11,fit.12,fit.13,fit.14,fit.15,fit.16)

more_deprived1_2012.aictab<-AICcmodavg::aictab(cand.set = models)
```

```
Warning in aictab.AICgls(cand.set = models):
Model names have been supplied automatically in the table
```

```
Warning in aictab.AICgls(cand.set = models):
Check model structure carefully as some models may be redundant
```

```
more_deprived1_2012.aictab
```

```
Model selection based on AICc:

       K   AICc Delta_AICc AICcWt Cum.Wt      LL
Mod10 11 449.18       0.00   0.30   0.30 -212.05
Mod3  11 450.13       0.95   0.19   0.48 -212.53
Mod11 12 451.07       1.89   0.12   0.60 -211.70
Mod4  12 451.98       2.80   0.07   0.67 -212.16
Mod8  18 452.28       3.10   0.06   0.73 -203.81
Mod1  10 452.41       3.23   0.06   0.79 -214.94
Mod2  10 452.41       3.23   0.06   0.85 -214.94
Mod12 13 452.94       3.76   0.05   0.90 -211.30
Mod14 16 453.06       3.89   0.04   0.94 -207.17
Mod5  13 453.71       4.53   0.03   0.97 -211.69
Mod13 14 455.39       6.22   0.01   0.99 -211.17
Mod6  14 456.20       7.03   0.01   0.99 -211.57
Mod15 18 457.77       8.59   0.00   1.00 -206.55
Mod7  16 460.19      11.01   0.00   1.00 -210.74
Mod16 22 463.84      14.66   0.00   1.00 -203.17
Mod9  22 464.13      14.95   0.00   1.00 -203.32
```

Keep the best model and delete the others

```
more_deprived1_2012.final.gls<-fit.10
rm(fit.01,fit.02,fit.03,fit.04,fit.05,fit.06,fit.07,fit.08,fit.09,fit.10,
   fit.11,fit.12,fit.13,fit.14,fit.15,fit.16,models)
```

Make table of results

```
more_deprived1_2012.gls.results.table<-gls.table.maker(more_deprived1_2012.final.gls)
more_deprived1_2012.gls.results.table
```

variable	Value	Std.Error	t-value	p-value
(Intercept)	15.95529	0.78553	20.31157	0.00000
time_months	0.06115	0.02336	2.61805	0.01039
announcement.trend	-0.08665	0.06349	-1.36486	0.17574
September	7.67773	0.88462	8.67909	0.00000
October	3.75024	0.88505	4.23734	0.00006
November	3.73391	0.82826	4.50814	0.00002
April	-4.40253	0.77437	-5.68530	0.00000
August	-8.06294	0.82712	-9.74820	0.00000
imp.trend	-0.03398	0.10132	-0.33533	0.73817

Get the fitted values from the model

```
more_deprived1_2012$predicted<-predict(more_deprived1_2012.final.gls)

tmp<-as.data.frame(predictSE.gls(
  mod = more_deprived1_2012.final.gls,newdata = more_deprived1_2012))
tmp$ci.min<-tmp$fit-(1.96*tmp$se.fit)
tmp$ci.max<-tmp$fit+(1.96*tmp$se.fit)
tmp<-select(tmp,ci.min,ci.max)

more_deprived1_2012$ci.min<-tmp$ci.min
more_deprived1_2012$ci.max<-tmp$ci.max
rm(tmp)
```

Add Counterfactuals 1 and 2 to the data table

```
more_deprived1_2012$cf<-NA

for(i in 1:nrow(more_deprived1_2012)){more_deprived1_2012$cf[i]<-
  counterfactual.function(i=i,
    df = more_deprived1_2012,
    model = more_deprived1_2012.final.gls)}

more_deprived1_2012$cf2<-NA

for(i in 1:nrow(more_deprived1_2012)){more_deprived1_2012$cf2[i]<-
  counterfactual2.function(i=i,
    df = more_deprived1_2012,
    model = more_deprived1_2012.final.gls)}
```

Chart the ITS result

```
more_deprived1_2012.its.chart<-its.model.plot(
  df = more_deprived1_2012,
    model = more_deprived1_2012.final.gls,points = FALSE,
    ylim.low = 0,ylim.high = 40, its.title="IMD 2 - More Deprived",
    its.ytitle = "Incidence rates per 100,000 population" )
more_deprived1_2012.its.chart
```

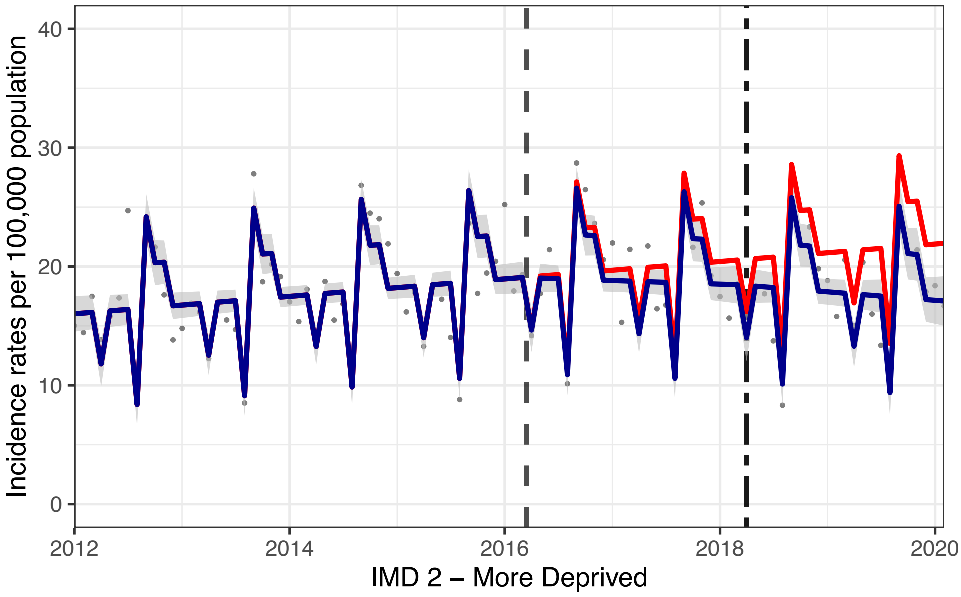

Get values of changes compared to counterfactual 1 (no intervention)

Note: The absolute difference takes no account for the confidence intervals on the counterfactual and is based only on the mean counterfactual values.

```
more_deprived1_2012.change.absolute <-
  get.predicted.anyweek(df = more_deprived1_2012,
                        absrel = "abs",pos = 98)
more_deprived1_2012.change.absolute
```

```
[1] "-4.85, 95% CI -2.77,-6.94) | -22.12%, 95% CI -31.6%,-12.63%)  "
```

Get values of changes compared to counterfactual 2 (no implementation)

Note: The absolute difference takes no account for the confidence intervals on the counterfactual and is based only on the mean counterfactual values.

```
more_deprived1_2012.change.absolute.cf2 <-
  get.predicted.anyweek.cf2(df = more_deprived1_2012,
                            absrel = "abs",pos = 98)
more_deprived1_2012.change.absolute.cf2
```

```
[1] "-0.78, 95% CI 1.3,-2.86) | -4.37%, 95% CI -16.02%,7.28%)  "
```

Calculate mean counts/ 100,000 persons/per month for pre and post-announceent

```
more_deprived1_2012$phase<-"pre_announcement"
more_deprived1_2012$phase[which(more_deprived1_2012$measurement_date>= "2016-03-01")]<-
"post_announcement"

more_deprived1_2012 %>%
  group_by (phase) %>%
  summarise (mean = mean (admiss_quint_prop, na.rm =T),
             (sd = sd (admiss_quint_prop, na.rm =T)))
```

```
# A tibble: 2 x 3
  phase              mean `(sd = sd(admiss_quint_prop, na.rm = T))`
  <chr>             <dbl>                                     <dbl>
1 post_announcement  18.7                                      4.47
2 pre_announcement   17.6                                      4.39
```

Asthma Analysis (IMD-1 - Most Deprived)

R code

- Code : Nina Rogers (Nina.Rogers@mrc-epid.cam.ac.uk)
- Code review : Chrissy h. Roberts (chrissy.roberts@LSHTM.ac.uk)

Data Prep and Functions Load

```
source("001_asthma_data_prep_age_sep.R")
source("007_asthma_deprivation_functions.R")
```

Filter the main Age-specific ITS dataset

```
most_deprived5 <-  by_sep_asthma %>%
  filter(Social_deprivation_quintile5 == "Most Deprived 20%")
```

Chart admissions data

This provides a simple eyeball test of the data trend across time.

```
 ggplot(most_deprived5, aes(x=measurement_date, y=admiss_quint_prop )) +
    geom_smooth() +
    xlab("")+
    ylab("Admissions per 100,000 population, per month")
```

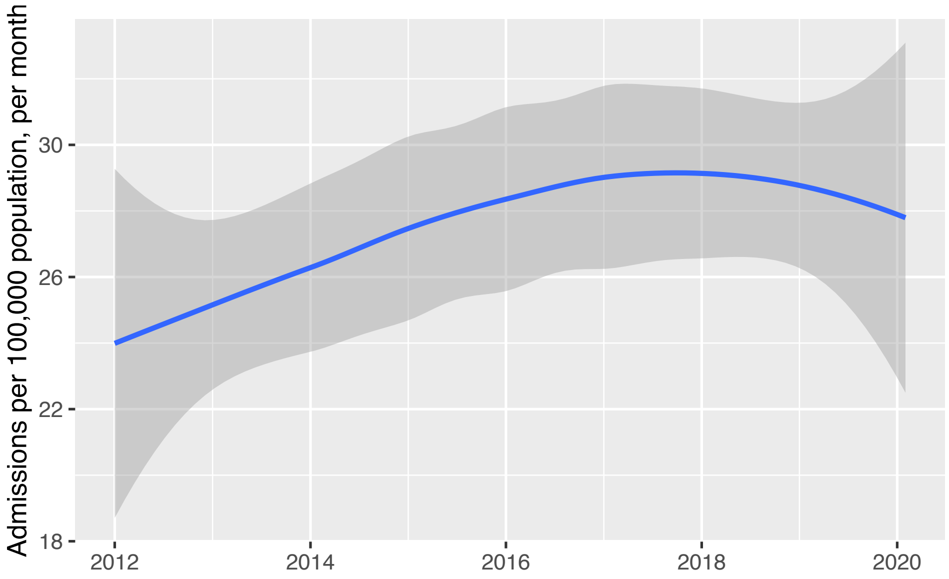

Create the time and intervention variables

This includes definition of time_months (study time), announcement.trend (change in trajectory post announcement) and imp.trend (change in trajectort post implementation).

```
most_deprived5$time_months <-c(1:98)
most_deprived5$announcement.trend <- c(rep(0,51),1:47)
most_deprived5$imp.trend <- c(rep(0,75),1:23)
most_deprived5 <- most_deprived5 %>% select(measurement_date, time_months, everything())
```

Run OLS analysis

```
most_deprived5.ols<-model_ols(df = most_deprived5)
summary(most_deprived5.ols)
```

```
Call:
lm(formula = admiss_quint_prop ~ time_months + announcement.trend +
    September + October + November + April + August + imp.trend,
    data = df)

Residuals:
    Min      1Q  Median      3Q     Max
-7.7555 -2.2677 -0.1276  1.4911 14.2949

Coefficients:
                    Estimate Std. Error t value Pr(>|t|)
(Intercept)         23.65019    0.97063  24.366  < 2e-16 ***
time_months          0.07753    0.02849   2.722  0.00782 **
announcement.trend  -0.03171    0.07726  -0.410  0.68250
September           14.22737    1.29618  10.976  < 2e-16 ***
October              6.43656    1.29662   4.964 3.30e-06 ***
November             6.30142    1.29729   4.857 5.06e-06 ***
April               -5.34981    1.29733  -4.124 8.36e-05 ***
August             -11.91200    1.29597  -9.192 1.51e-14 ***
imp.trend           -0.14506    0.12397  -1.170  0.24509
---
Signif. codes:  0 '***' 0.001 '**' 0.01 '*' 0.05 '.' 0.1 ' ' 1

Residual standard error: 3.436 on 89 degrees of freedom
Multiple R-squared:  0.7837,    Adjusted R-squared:  0.7643
F-statistic: 40.32 on 8 and 89 DF,  p-value: < 2.2e-16
```

Run Durbin-Watson Test

```
most_deprived5.dwt<-model_dwt(most_deprived5.ols)
most_deprived5.dwt
```

```
lag Autocorrelation D-W Statistic p-value
  1     0.048894097      1.895377   0.438
  2    -0.095662015      2.177629   0.478
  3    -0.029841579      2.042795   0.884
  4     0.033961733      1.910812   0.726
  5    -0.064669294      2.102466   0.504
  6    -0.114694870      2.199991   0.212
  7    -0.045490155      1.867058   0.760
  8    -0.078077854      1.912996   0.908
  9    -0.084363272      1.914553   0.846
 10    -0.033938211      1.813058   0.864
 11    -0.047227993      1.825162   0.970
 12     0.008850676      1.678292   0.282
Alternative hypothesis: rho[lag] != 0
```

Chart acf of ols

```
graph.ols.acf.plots(model = most_deprived5.ols)
```

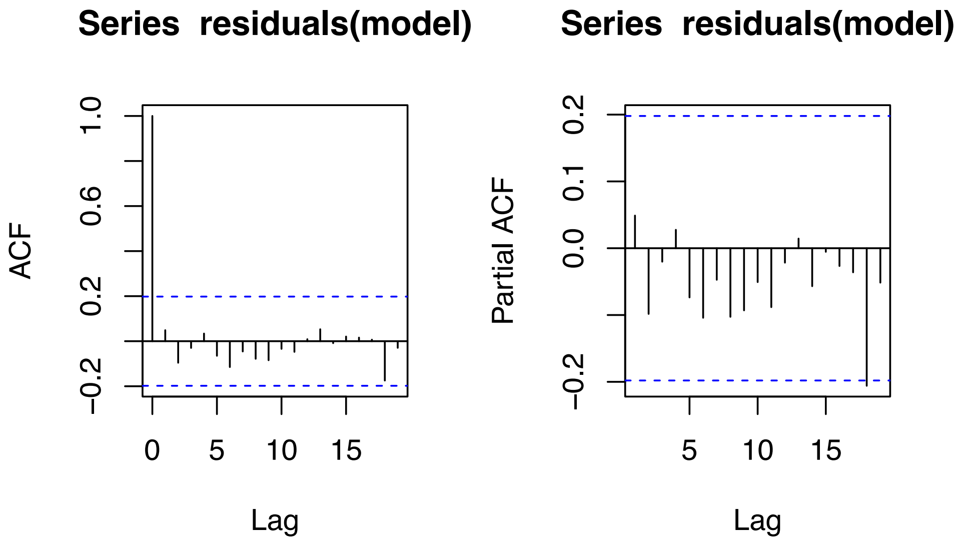

Run gls models with a variety of values of p and q

Run a variety of values here to provide belt and braces. Using values 1,2,3,4,6,8 and 12, allows for various cyclical calendar-relevant lags. fit.01 provides a linear relationship between x and y with no lag, whilst fit.02 provides a time-squared function without lag.

```
fit.01<-gls.modeller(df = most_deprived5,
                     timesquared= FALSE,timecubed= FALSE,p=NULL,q=NULL)
fit.02<-gls.modeller(df = most_deprived5,
                     timesquared= TRUE,timecubed= FALSE,p=NULL,q=NULL)
fit.03<-gls.modeller(df = most_deprived5,
                     timesquared= FALSE,timecubed= FALSE,p=1,q=NULL)
fit.04<-gls.modeller(df = most_deprived5,
                     timesquared= FALSE,timecubed= FALSE,p=2,q=NULL)
fit.05<-gls.modeller(df = most_deprived5,
                     timesquared= FALSE,timecubed= FALSE,p=3,q=NULL)
fit.06<-gls.modeller(df = most_deprived5,
                     timesquared= FALSE,timecubed= FALSE,p=4,q=NULL)
fit.07<-gls.modeller(df = most_deprived5,
                     timesquared= FALSE,timecubed= FALSE,p=6,q=NULL)
fit.08<-gls.modeller(df = most_deprived5,
                     timesquared= FALSE,timecubed= FALSE,p=8,q=NULL)
fit.09<-gls.modeller(df = most_deprived5,
                     timesquared= FALSE,timecubed= FALSE,p=12,q=NULL)


fit.10<-gls.modeller(df = most_deprived5,
                     timesquared= FALSE,timecubed= FALSE,p=NULL,q=1)
fit.11<-gls.modeller(df = most_deprived5,
                     timesquared= FALSE,timecubed= FALSE,p=NULL,q=2)
fit.12<-gls.modeller(df = most_deprived5,
                     timesquared= FALSE,timecubed= FALSE,p=NULL,q=3)
fit.13<-gls.modeller(df = most_deprived5,
                     timesquared= FALSE,timecubed= FALSE,p=NULL,q=4)
fit.14<-gls.modeller(df = most_deprived5,
                     timesquared= FALSE,timecubed= FALSE,p=NULL,q=6)
fit.15<-gls.modeller(df = most_deprived5,
                     timesquared= FALSE,timecubed= FALSE,p=NULL,q=8)
fit.16<-gls.modeller(df = most_deprived5,
                     timesquared= FALSE,timecubed= FALSE,p=NULL,q=12)
```

Compare the AIC values for various possible fits

```
models<-list(fit.01,fit.02,fit.03,fit.04,fit.05,fit.06,fit.07,fit.08,fit.09,fit.10,
             fit.11,fit.12,fit.13,fit.14,fit.15,fit.16)

most_deprived5.aictab<-AICcmodavg::aictab(cand.set = models)
```

```
Warning in aictab.AICgls(cand.set = models):
Model names have been supplied automatically in the table
```

```
Warning in aictab.AICgls(cand.set = models):
Check model structure carefully as some models may be redundant
```

```
most_deprived5.aictab
```

```
Model selection based on AICc:

       K   AICc Delta_AICc AICcWt Cum.Wt      LL
Mod1  10 533.10       0.00   0.31   0.31 -255.29
Mod2  10 533.10       0.00   0.31   0.63 -255.29
Mod10 11 535.31       2.21   0.10   0.73 -255.12
Mod3  11 535.38       2.28   0.10   0.83 -255.16
Mod4  12 536.55       3.45   0.06   0.89 -254.44
Mod11 12 536.81       3.71   0.05   0.94 -254.57
Mod14 16 538.42       5.32   0.02   0.96 -249.85
Mod12 13 539.06       5.96   0.02   0.97 -254.37
Mod5  13 539.20       6.10   0.01   0.99 -254.43
Mod6  14 541.57       8.47   0.00   0.99 -254.25
Mod13 14 541.62       8.52   0.00   1.00 -254.28
Mod15 18 543.48      10.38   0.00   1.00 -249.41
Mod7  16 545.20      12.10   0.00   1.00 -253.24
Mod16 22 546.72      13.62   0.00   1.00 -244.61
Mod8  18 550.63      17.52   0.00   1.00 -252.98
Mod9  22 560.02      26.92   0.00   1.00 -251.26
```

Keep the best model and delete the others

```
most_deprived5.final.gls<-fit.01
rm(fit.01,fit.02,fit.03,fit.04,fit.05,fit.06,fit.07,fit.08,fit.09,fit.10,
   fit.11,fit.12,fit.13,fit.14,fit.15,fit.16,models)
```

Make table of results

```
most_deprived5.gls.results.table<-gls.table.maker(most_deprived5.final.gls)
most_deprived5.gls.results.table
```

variable	Value	Std.Error	t-value	p-value
(Intercept)	23.65019	0.97063	24.36570	0.00000
time_months	0.07753	0.02849	2.72153	0.00782
announcement.trend	-0.03171	0.07726	-0.41040	0.68250
September	14.22737	1.29618	10.97635	0.00000
October	6.43656	1.29662	4.96409	0.00000
November	6.30142	1.29729	4.85738	0.00001
April	-5.34981	1.29733	-4.12372	0.00008
August	-11.91200	1.29597	-9.19160	0.00000
imp.trend	-0.14506	0.12397	-1.17008	0.24509

Get the fitted values from the model

```
most_deprived5$predicted<-predict(most_deprived5.final.gls)

tmp<-as.data.frame(predictSE.gls(mod = most_deprived5.final.gls,newdata = most_deprived5))
tmp$ci.min<-tmp$fit-(1.96*tmp$se.fit)
tmp$ci.max<-tmp$fit+(1.96*tmp$se.fit)
tmp<-select(tmp,ci.min,ci.max)

most_deprived5$ci.min<-tmp$ci.min
most_deprived5$ci.max<-tmp$ci.max
rm(tmp)
```

Add Counterfactuals 1 and 2 to the data table

```
most_deprived5$cf<-NA

for(i in 1:nrow(most_deprived5)){most_deprived5$cf[i]<-counterfactual.function(i=i,
                                                 df = most_deprived5,
                                                 model = most_deprived5.final.gls)}

most_deprived5$cf2<-NA

for(i in 1:nrow(most_deprived5)){most_deprived5$cf2[i]<-counterfactual2.function(i=i,
                                                 df = most_deprived5,
                                                 model = most_deprived5.final.gls)}
```

Chart the ITS result

```
most_deprived5.its.chart<-its.model.plot(
  df = most_deprived5,
  model = most_deprived5.final.gls,points = FALSE,
  ylim.low = 0,
  ylim.high = 50,
  its.title="IMD 1 - Most Deprived",
  its.ytitle = "Incidence rates per 100,000 population" )
most_deprived5.its.chart
```

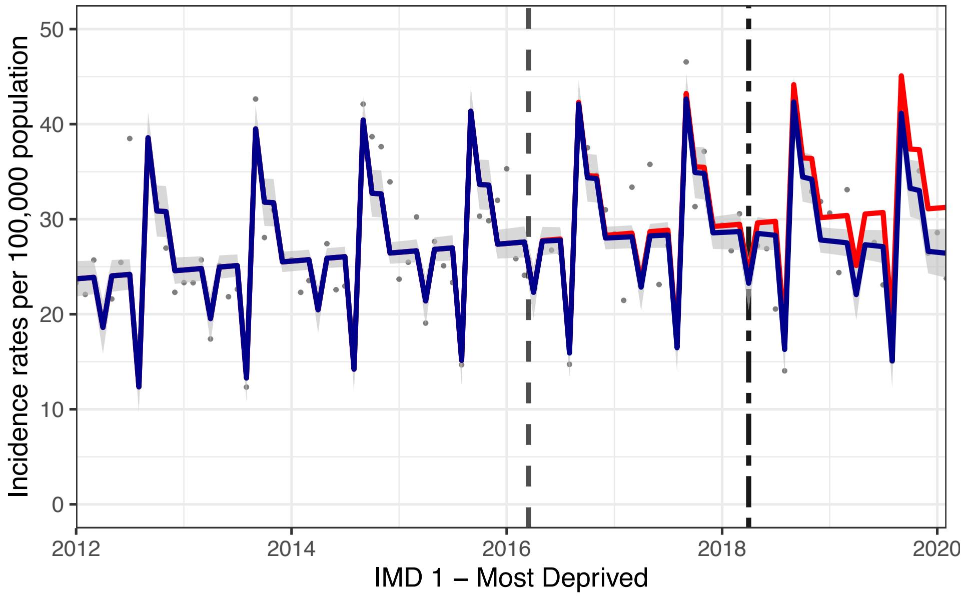

Get values of changes compared to counterfactual 1 (no intervention)

Note: The absolute difference takes no account for the confidence intervals on the counterfactual and is based only on the mean counterfactual values.

```
most_deprived5.change.absolute <-
  get.predicted.anyweek(df = most_deprived5,
  absrel = "abs",pos = 98)
most_deprived5.change.absolute
```

```
[1] "-4.83, 95% CI -2.25,-7.41) | -15.45%, 95% CI -23.7%,-7.19%)  "
```

Get values of changes compared to counterfactual 2 (no implementation)

Note: The absolute difference takes no account for the confidence intervals on the counterfactual and is based only on the mean counterfactual values.

```
most_deprived5.change.absolute.cf2 <-
  get.predicted.anyweek.cf2(
    df = most_deprived5,
    absrel = "abs",pos = 98)
most_deprived5.change.absolute.cf2
```

```
[1] "-3.34, 95% CI -0.76,-5.92) | -11.21%, 95% CI -19.88%,-2.54%)  "
```

Calculate mean counts/ 100,000 persons/per month for pre and post-announceent

```
most_deprived5$phase<-"pre_announcement"
most_deprived5$phase[which(most_deprived5$measurement_date>= "2016-03-01")]<-
  "post_announcement"

most_deprived5 %>% group_by (phase) %>%
  summarise (mean = mean (admiss_quint_prop, na.rm =T),
             (sd = sd (admiss_quint_prop, na.rm =T)))
```

```
# A tibble: 2 x 3
  phase              mean `(sd = sd(admiss_quint_prop, na.rm = T))`
  <chr>             <dbl>                                     <dbl>
1 post_announcement  28.6                                      7.02
2 pre_announcement   26.4                                      7.03
```

Asthma Analysis

R code

The following R script processes and prepares the data set for the analyses shown above.

- Code : Nina Rogers (Nina.Rogers@mrc-epid.cam.ac.uk)
- Code review : Chrissy h. Roberts (chrissy.roberts@LSHTM.ac.uk)

```
##############################################################################
# The UK Soft Drinks Industry Levy and childhood hospital admissions for asthma in England: interrupted time series analysis (2012-2020)
# Rogers et al.
# Code : Nina Rogers [Nina.Rogers@mrc-epid.cam.ac.uk]
# Code review : Chrissy h Roberts [chrissy.roberts@lshtm.ac.uk]
##############################################################################
# Data preparation script
#
# This script processes and analyzes asthma diagnosis data using R,
# focusing on admissions data across different months and demographic groups
##############################################################################



################################################################################
# LIBRARIES
################################################################################

{
  library(ggplot2)
  library(reshape2)
  library(data.table)
  library(car)
  library(nlme)
  library(sandwich)
  library(lmtest)
  library(dplyr)
  library(summarytools)
  library(lubridate)
  library(flextable)
  library (gmodels)
  library(tidyverse)
  library(AICcmodavg)
  library (patchwork)
  library("readxl")
}


################################################################################
# READ DATA
# THE DATASET IS NOT PUBLISHED WITH THE PAPER AS IT IS CONTROLLED BY NHS DIGITAL
#
################################################################################

asthma_primarydiagnosis <- read_excel("data/dental_primarydiagnosis.xlsx")


################################################################################
# CONVERT MONTH DATA IN TO A BINARY MATRIX
################################################################################
{
asthma_primarydiagnosis$January <- ifelse(asthma_primarydiagnosis$Month == "1" , 1,0)
asthma_primarydiagnosis$February <- ifelse(asthma_primarydiagnosis$Month == "2" , 1,0)
asthma_primarydiagnosis$March <- ifelse(asthma_primarydiagnosis$Month == "3" , 1,0)
asthma_primarydiagnosis$April <- ifelse(asthma_primarydiagnosis$Month == "4" , 1,0)
asthma_primarydiagnosis$May <- ifelse(asthma_primarydiagnosis$Month == "5" , 1,0)
asthma_primarydiagnosis$June <- ifelse(asthma_primarydiagnosis$Month == "6" , 1,0)
asthma_primarydiagnosis$July <- ifelse(asthma_primarydiagnosis$Month == "7" , 1,0)
asthma_primarydiagnosis$August <- ifelse(asthma_primarydiagnosis$Month == "8" , 1,0)
asthma_primarydiagnosis$September <- ifelse(asthma_primarydiagnosis$Month == "9" , 1,0)
asthma_primarydiagnosis$October <- ifelse(asthma_primarydiagnosis$Month == "10" , 1,0)
asthma_primarydiagnosis$November <- ifelse(asthma_primarydiagnosis$Month == "11" , 1,0)
asthma_primarydiagnosis$December <- ifelse(asthma_primarydiagnosis$Month == "12" , 1,0)
}
################################################################################
# Make data in format YMD and filter
#
# Filter multiple diagnosese to keep only Asthma data
# Set date range to include only 2011-12-01 to 2020-02-29
################################################################################

asthma_primarydiagnosis <- asthma_primarydiagnosis %>%
  mutate (
    measurement_date=str_c(Year,"-",Month,"-01"),
    measurement_date=as.Date(measurement_date),
    Social_deprivation_quintile5 = as.factor(Social_deprivation_quintile5)
  ) %>%
  filter(Age_Group =="B 5-9" |Age_Group =="C 10-14" | Age_Group =="D 15-18" ) %>%
  filter (Diagnosis_Description == "Asthma" , measurement_date < "2020-03-01", measurement_date > "2011-12-01")

################################################################################
# READ IN THE DENOMINATOR DATA FOR THE FULL COHORT
################################################################################
age05_18 <- read_csv("data/05_18_denom.csv")
age05_18 <- age05_18 %>%
  rename(
    measurement_date = dt,
    denominator = n
  )


################################################################################
# CONVERT THE DENOMINATOR DATA IN TO NUMBERS FOR SUBDIVISIONS
# QUINTILES FOR DEMOGRAPHICS ARE BY DEFINITION 1/5 TOTAL DENOMINATOR
# ASSUMPTION THAT SEX IS SPLIT IN TO 1/2
# SEX QUINTILES SPLITS ON ANY QUINTILE PLUS 1/2 DIVISION (i.e. 1/10s overall)
# INCLUDING SEX HERE IN CASE A FUTURE ANALYST WANTS TO DO A SEX BASED ANALYSIS
################################################################################

age05_18 <- age05_18 %>% mutate(
  denom_quint = denominator/5,
  denom_sex = denominator/2,
  denom_sex_quint = denominator/10,
  measurement_date = as.Date(measurement_date, format = "%d/%m/%Y")
)


######################################################################
# JOIN DIAGNOSIS DATA TO THE DENOMINATORS TO GET SOCIOECONOMIC POSITION DATA
######################################################################

sep.data <- left_join (asthma_primarydiagnosis, age05_18)
```

```
######################################################################
# CREATE ITS DATASET FOR ASTHMA CASES BY SOCIOECONOMIC POSITION
######################################################################

by_sep_asthma <- sep.data %>%
  group_by(measurement_date, Social_deprivation_quintile5) %>%
  reframe(
    admissions_no = sum(Admissions, na.rm=T),
    denom_quint,
    admiss_quint_prop = (admissions_no * 100000)/denom_quint,
    December,
    January,
    February,
    March,
    April,
    May,
    June,
    July,
    August,
    September,
    October,
    November
  ) %>%
  filter(measurement_date < "2020-03-01", measurement_date > "2011-12-01") %>%
  distinct()


######################################################################
# Draw a  basic plot of admission COUNTS across time, uncorrected for denominators
######################################################################
(ggplot(by_sep_asthma, aes(x=measurement_date, y=admissions_no, color=Social_deprivation_quintile5 )) +
    geom_smooth() +
    xlab("")
)
ggsave("outputs/admissions_by_deprived.its.chart.pdf",width = 10,height = 8,dpi = 300)


######################################################################
# IMPORT AND ALIGN DENOMINATORS FOR THE 05-09 AGE GROUP
######################################################################

age05_09 <- read_csv("data/05_09_denom.csv") %>%
  rename(
    measurement_date = dt,
    denominator = n
  ) %>%
  mutate(
    Age_Group = "B 5-9",
    measurement_date = as.Date(measurement_date, format = "%d/%m/%Y")
  )

######################################################################
# IMPORT AND ALIGN DENOMINATORS FOR THE 10-14 AGE GROUP
######################################################################

age10_14 <- read_csv("data/10_14_denom.csv") %>%
  rename(
    measurement_date = dt,
    denominator = n
  ) %>%
  mutate(
    Age_Group = "C 10-14",
    measurement_date = as.Date(measurement_date, format = "%d/%m/%Y")
  )

######################################################################
# IMPORT AND ALIGN DENOMINATORS FOR THE 15-18 AGE GROUP
######################################################################

age15_18 <- read_csv("data/15_18_denom.csv")  %>%
  rename(
    measurement_date = dt,
    denominator = n
  ) %>%
  mutate(
    Age_Group = "D 15-18",
    measurement_date = as.Date(measurement_date, format = "%d/%m/%Y")
  )

######################################################################
# BIND ALL THE AGE GROUP DATA TOGETHER TO MAKE A TIDY TABLE
######################################################################
age_grps<- bind_rows(age05_09, age10_14, age15_18, age05_18)


age.data <- left_join (asthma_primarydiagnosis, age_grps, relationship="many-to-many")

######################################################################
# CREATE A TABLE OF ITS DATA, SPLIT BY AGE GROUPS
######################################################################

by_age_asthma <- age.data %>%
  group_by(measurement_date, Age_Group) %>%
  reframe(
    admissions_no = sum(Admissions, na.rm=T),
    denominator,
    admission_prop = (admissions_no * 100000)/denominator,
    December,
    January,
    February,
    March,
    April,
    May,
    June,
    July,
    August,
    September,
    October,
    November
  ) %>%
  filter(measurement_date < "2020-03-01", measurement_date > "2011-12-01") %>%

  distinct()


######################################################################
```

```
# DRAW A BASIC CHART TO SHOW ADMISSIONS PER 100,000 POPULATION, PER MONTH
####################################################################

(ggplot(by_age_asthma, aes(x=measurement_date, y=admission_prop, color=Age_Group )) +
    geom_smooth() +
    xlab("")+
   ylab("Admissions per 100,000 population, per month")

)
ggsave("outputs/admissions_by_age.its.chart.pdf",width = 10,height = 8,dpi = 300)




####################################################################
# CREATE AN ITS DATASET FOR THE WHOLE POPULATION (05-18)
# WITH DIVISIONS BY SEX AND IMD QUINTILE
####################################################################

age05_18 <- read_csv("data/05_18_denom.csv")  %>%
  rename(
    measurement_date = dt,
    denominator = n
  ) %>%
  mutate(
    Age_Group = "E 05-18",
    measurement_date = as.Date(measurement_date, format = "%d/%m/%Y")
  )

whole.data <- left_join (asthma_primarydiagnosis, select(age05_18,measurement_date,denominator))%>%
                  mutate(Age_Group="E 5-18")


####################################################################
# CREATE ITS DATA FOR SOCIOECONOMIC POSITION (AGE 05-18)
####################################################################

by_whole_asthma <- whole.data %>%
  group_by(measurement_date) %>%
  reframe(
    admissions_no = sum(Admissions, na.rm=T),
    admission_prop = (admissions_no * 100000)/denominator,
    December,
    January,
    February,
    March,
    April,
    May,
    June,
    July,
    August,
    September,
    October,
    November
  ) %>%
  filter(measurement_date < "2020-03-01", measurement_date > "2011-12-01") %>%

  distinct()
```

Asthma Analysis

R code

The following R script creates system functions for performing the analyses on age groups

- Code : Nina Rogers (Nina.Rogers@mrc-epid.cam.ac.uk)
- Code review : Chrissy h. Roberts (chrissy.roberts@LSHTM.ac.uk)

```
###############################################################################
# The UK Soft Drinks Industry Levy and childhood hospital admissions for asthma in England: interrupted time series analysis (2012-2020)
# Rogers et al.
# Code : Nina Rogers [Nina.Rogers@mrc-epid.cam.ac.uk]
# Code review : Chrissy h Roberts [chrissy.roberts@lshtm.ac.uk]
###############################################################################
# Functions to support time series analysis
# This version is for analysis of AGE differences
###############################################################################


#################################################################################
# LIBRARIES
#################################################################################

{
  library(ggplot2) # For data visualization.
  library(reshape2) # For reshaping data.
  library(data.table) # For efficient data manipulation.
  library(car) # For Companion to Applied Regression, includes Durbin-Watson test.
  library(nlme) # For fitting Generalized Least Squares (GLS) models.
  library(sandwich) # For robust statistical estimation.
  library(lmtest) # For testing linear regression models, includes robust standard errors.
  library(dplyr) # For data manipulation and transformation.
  library(summarytools) # For descriptive statistics and data summaries.
  library(lubridate) # For date-time manipulation.
  library(flextable) # For creating flexible and complex tables.
  library(gmodels) # For various model fitting functions.
  library(tidyverse) # For an opinionated collection of data science packages.
  library(AICcmodavg) # For model selection and multimodel inference based on AICc.
  library(patchwork) # For combining multiple ggplot objects into a single plot.

}

###############################################################################
###############################################################################
# Function Definitions
###############################################################################
###############################################################################

###############################################################################
# OLS Model Function
#
# Purpose: Fits an Ordinary Least Squares (OLS) regression model to the data, predicting admission_prop from time, certain months, and trend variables.
# Input: df, a data frame containing the variables of interest.
# Output: An object of class lm, representing the fitted linear model.
#
# For this study, the months of Sept, Oct, Nov, April and August are significant
###############################################################################

model_ols<-function(df){
  fit<-
    lm(admission_prop ~ time_months  + announcement.trend  + September + October + November + April + August +  imp.trend,data = df)
  return(fit)
}

###############################################################################
# Durbin-Watson Test Function
#
# Purpose: Applies the Durbin-Watson test to a model to assess the presence of autocorrelation in the residuals.
# Input: model, a fitted model object.
# Output: The result of the Durbin-Watson test.
#
# For this study, the months of Sept, Oct, Nov, April and August are significant
###############################################################################

model_dwt<-function(model){dwt (model, max.lag =20, alternative = "two.sided")}



#########################################################################
# Residual Plot Function
#
# Purpose: Generates a scatter plot of OLS model residuals over time.
# Input: df (data frame), model (fitted model object), and color1 (color for the points).
# Output: A plot displaying the residuals of the OLS model.
###############################################################################

graph.ols.residuals<-function(df,model,color1="red"){
  plot (df$time_months[1:74],
        residuals(model)[1:734],
        type='o',
        pch = 20,
        xlab = 'time_months',
        ylab = 'ols residuals',
        col = color1)
  abline(h=0, lty=2)}

####################################################################
# ACF Plot Function
#
# Purpose: Displays autocorrelation and partial autocorrelation plots for the residuals of a fitted model.
# Input: model, a fitted model object.
# Output: Side-by-side ACF and PACF plots for model diagnostics.
###################################################################

graph.ols.acf.plots<-function(model){
  #set plotting to 2 records on one page

  par(mfrow=c(1,2))

  acf(residuals(model))
  acf(residuals(model), type = 'partial')

  #plot one record on one page
  par(mfrow=c(1,1))
}



###############################################################################
```

```
# GLS Model Fitting Function
#
# Purpose: Fits a Generalized Least Squares (GLS) model to the data with options for including squared or cubed time terms and specifying ARMA(p,q)
correlation structures.
# Input: df (data frame with data), timesquared and timecubed (logical flags for including time squared/cubed terms), and p, q (parameters for ARMA
correlation structure).
# Output: A fitted gls model.
#############################################################################

gls.modeller<-function(df,timesquared=FALSE,timecubed=FALSE,p=NULL,q=NULL){


  # for cases where p and q are null
  if(timesquared==F & timecubed==F & is.null(p) & is.null(q)) { a<- gls(admission_prop ~ time_months +  announcement.trend +  September + October +
November + April + August + imp.trend, data = df,method="ML")}
  if(timesquared==T & timecubed==F & is.null(p) & is.null(q)) { a<- gls(admission_prop ~ time_months +  announcement.trend +  September + October +
November + April + August + imp.trend, data = df,method="ML")}
  if(timesquared==F & timecubed==T & is.null(p) & is.null(q)) { a <-gls(admission_prop ~ time_months + announcement.trend +   September + October +
November + April + August + imp.trend, data = df,method="ML")}

  # for cases where p is provided
  if(timesquared==F & timecubed==F & !is.null(p) & is.null(q)) { a<- gls(admission_prop ~ time_months +  announcement.trend +  September + October +
November + April + August + imp.trend, data = df,correlation= corARMA(p=p, form = ~ time_months),method="ML")}
  if(timesquared==T & timecubed==F & !is.null(p) & is.null(q)) { a<- gls(admission_prop ~ time_months +  announcement.trend  + September + October +
November + April + August + imp.trend + time_months^2, data = df,correlation= corARMA(p=p, form = ~ time_months),method="ML")}
  if(timesquared==T & timecubed==F & !is.null(p) & is.null(q)) { a<- gls(admission_prop ~ time_months +  announcement.trend  +  September + October +
November + April + August + imp.trend + time_months^3 , data = df,correlation= corARMA(p=p, form = ~ time_months),method="ML")}

  #for cases where q is provided
  if(timesquared==F & timecubed==F & is.null(p) & !is.null(q)) { a<- gls(admission_prop ~ time_months  + announcement.trend +  September + October +
November + April + August + imp.trend , data = df,correlation= corARMA(q=q, form = ~ time_months),method="ML")}
  if(timesquared==T & timecubed==F & is.null(p) & !is.null(q)) { a<- gls(admission_prop ~ time_months  + announcement.trend +  September + October +
November + April + August + imp.trend + time_months^2, data = df,correlation= corARMA(q=q, form = ~ time_months),method="ML")}
  if(timesquared==F & timecubed==T & is.null(p) & !is.null(q)) { a<- gls(admission_prop ~ time_months  + announcement.trend + September + October +
November + April + August + imp.trend + time_months^3 , data = df,correlation= corARMA(q=q, form = ~ time_months),method="ML")}

  # for cases where both p and q are provided
  if(timesquared==F & timecubed==F & !is.null(p) & !is.null(q)) { a<- gls(admission_prop ~ time_months  + announcement.trend + September + October +
November + April + August + imp.trend,  data = df,correlation= corARMA(p=p, form = ~ time_months),method="ML")}
  if(timesquared==T & timecubed==F & !is.null(p) & !is.null(q)) { a<- gls(admission_prop ~ time_months  + announcement.trend +  September + October +
November + April + August + imp.trend + time_months^2, data = df,correlation= corARMA(p=p,q=q, form = ~ time_months),method="ML")}
  if(timesquared==F & timecubed==T & !is.null(p) & !is.null(q)) { a<- gls(admission_prop ~ time_months  + announcement.trend +  September + October +
November + April + August + imp.trend + time_months^3 , data = df,correlation= corARMA(p=p,q=q, form = ~ time_months),method="ML")}

  return(a)
}


#############################################################################
# GLS Results Table Function
#
# Purpose: Creates a formatted table summarizing the results of a GLS model.
# Input: df, a fitted gls model, and caption, a string for the table caption.
# Output: A flextable object displaying the model's coefficient estimates, standard errors, t-values, and p-values.
#############################################################################

gls.table.maker<-function(df,caption=""){

  df.results<-round(as.data.frame(summary(df)$tTable),5)
  df.results$variable<-rownames(df.results)
  df.results<-select(df.results,"variable","Value","Std.Error","t-value","p-value")
  flextable(df.results)
  df.results<-flextable(df.results)
  return(df.results)


}

###############################################################################
###############################################################################
# Counterfactual Function
#
# Purpose: These functions (counterfactual.function) computes estimated values (counterfactuals) based on the model's coefficients, reflecting
scenarios without certain interventions.
# Input: Indices for specific observations, the data frame, and a fitted model.
# Output: The counterfactual estimates.
#
# Scenario : CF is no intervention
###############################################################################

counterfactual.function<-function(i,df,model){
  a<- sum(
    (model$coef[1]),                        #add intercept for admission_prop
    (model$coef[2] * df$time_months[i]),   #add effect of drinks across time
    (model$coef[4] * df$September[i]),     #add effect of September
    (model$coef[5] * df$October[i]),       #add effect of October
    (model$coef[6] * df$November[i]),      #add effect of November
    (model$coef[7] * df$April[i]),         #add effect of April
    (model$coef[8] * df$August[i])         #add effect of August
        )
    as.numeric(a)
}


###############################################################################
###############################################################################
# Counterfactual Function
#
# Purpose: These functions (counterfactual.function) computes estimated values (counterfactuals) based on the model's coefficients, reflecting
scenarios without certain interventions.
# Input: Indices for specific observations, the data frame, and a fitted model.
# Output: The counterfactual estimates.
#
# Scenario : CF is an announcement, but law does not come in to force
# i.e. there was an announcement, but no 'implementation'
###############################################################################

counterfactual2.function<-function(i,df,model){
  a<- sum(

    (model$coef[1]),    #add intercept for admission_prop
    (model$coef[2] * df$time_months[i]),#add effect of drinks across time
    (model$coef[4] * df$September[i]),#add effect of september
```

```
(model$coef[5] * df$October[i]),#add effect of october
(model$coef[6] * df$November[i]),#add effect of nov
(model$coef[7] * df$April[i]),#add effect of April
(model$coef[8] * df$August[i]),#add effect of August
(model$coef[3]*df$announcement.trend[i])#add observed change drinks across time after announcement
    )
as.numeric(a)

}


################################################################################
# ITS Model Plot Function
#
# Purpose: Visualizes the fit of an Interrupted Time Series (ITS) model, including actual vs.
predicted values and confidence intervals.
# Input: df (data frame), model (fitted model object), visualization parameters, and titles.
# Output: A ggplot object showing the ITS analysis results.
################################################################################

its.model.plot<-function(df,model,ylim.low,ylim.high,points=TRUE, its.title ="", its.ytitle =""){
  a <- ggplot (df,aes(measurement_date,admission_prop))+
  geom_point(size=0.4,alpha=0.5)+
  geom_vline(xintercept = as.Date("2016-03-15","%Y-%m-%d"),linetype = 2,alpha=0.7, lwd = 1,  color =
  "black") +
  geom_vline(xintercept = as.Date("2018-04-01", "%Y-%m-%d"),linetype = 6 ,alpha=0.9, lwd = 1, color =
  "black") +
  geom_ribbon(aes(ymin=ci.min,ymax=ci.max,colour=NULL),fill="grey",alpha=0.6)+

  theme(legend.position = "none") +

  scale_x_date(expand = c(0, 0)) +
  ylim (ylim.low, ylim.high) +
  labs(x = its.title) +
  labs(y = its.ytitle) +
  theme_bw() +
  geom_line(data = df,aes(measurement_date,cf),lwd=1.0,lty=1,color="red") +
  geom_line(aes(measurement_date, predicted),lwd=1.0,color="dark blue")

return(a)


}


################################################################################
# Predicted Difference Functions
#
# Purpose : Functions  get.predicted.anyweek and get.predicted.anyweek.cf2 calculate the difference
between actual and counterfactual
# (or predicted) values at specified time points, useful for assessing the impact of interventions.
################################################################################


get.predicted.anyweek<-function(df,absrel="abs",pos){
  if(absrel=="abs"){
  x <- diff(c(df$cf[pos],df$predicted [pos]))
  x2<- diff(c(df$cf[pos],df$ci.min [pos]))
  x3<- diff(c(df$cf[pos],df$ci.max [pos]))
  x4<- round(100*(x/df$cf[pos]),2)
  x5<- round(100*(x2/df$cf[pos]),2)
  x6<- round(100*(x3/df$cf[pos]),2)

  x<-paste0(round(x,2),", 95% CI ",round(x3,2),",",round(x2,2),") | ", x4,"%, 95% CI ",x5,"%,",x6,"%)
  ")

}

return(x)


}


get.predicted.anyweek.cf2<-function(df,absrel="abs",pos){
  if(absrel=="abs"){
  x <- diff(c(df$cf2[pos],df$predicted [pos]))
  x2<- diff(c(df$cf2[pos],df$ci.min [pos]))
  x3<- diff(c(df$cf2[pos],df$ci.max [pos]))
  x4<- round(100*(x/df$cf2[pos]),2)
  x5<- round(100*(x2/df$cf2[pos]),2)
  x6<- round(100*(x3/df$cf2[pos]),2)

  x<-paste0(round(x,2),", 95% CI ",round(x3,2),",",round(x2,2),") | ", x4,"%, 95% CI ",x5,"%,",x6,"%)
  ")

}

return(x)
}
```

Asthma Analysis

R code

The following R script creates system functions for performing the analyses on deprivation groups

- Code : Nina Rogers (Nina.Rogers@mrc-epid.cam.ac.uk)
- Code review : Chrissy h. Roberts (chrissy.roberts@LSHTM.ac.uk)

```
#############################################################################
# The UK Soft Drinks Industry Levy and childhood hospital admissions for asthma in England: interrupted time series analysis (2012-2020)
# Rogers et al.
# Code : Nina Rogers [Nina.Rogers@mrc-epid.cam.ac.uk]
# Code review : Chrissy h Roberts [chrissy.roberts@lshtm.ac.uk]
#############################################################################
# Functions to support time series analysis
# This version is for analysis of DEPRIVATION differences
#############################################################################


###############################################################################
# LIBRARIES
###############################################################################

{
  library(ggplot2) # For data visualization.
  library(reshape2) # For reshaping data.
  library(data.table) # For efficient data manipulation.
  library(car) # For Companion to Applied Regression, includes Durbin-Watson test.
  library(nlme) # For fitting Generalized Least Squares (GLS) models.
  library(sandwich) # For robust statistical estimation.
  library(lmtest) # For testing linear regression models, includes robust standard errors.
  library(dplyr) # For data manipulation and transformation.
  library(summarytools) # For descriptive statistics and data summaries.
  library(lubridate) # For date-time manipulation.
  library(flextable) # For creating flexible and complex tables.
  library(gmodels) # For various model fitting functions.
  library(tidyverse) # For an opinionated collection of data science packages.
  library(AICcmodavg) # For model selection and multimodel inference based on AICc.
  library(patchwork) # For combining multiple ggplot objects into a single plot.

}

#############################################################################
#############################################################################
# Function Definitions
#############################################################################
#############################################################################

#############################################################################
# OLS Model Function
#
# Purpose: Fits an Ordinary Least Squares (OLS) regression model to the data, predicting admission_prop from time, certain months, and trend variables.
# Input: df, a data frame containing the variables of interest.
# Output: An object of class lm, representing the fitted linear model.
#
# For this study, the months of Sept, Oct, Nov, April and August are significant
#############################################################################

model_ols<-function(df){
  fit<-
    lm(  admiss_quint_prop ~ time_months  + announcement.trend + September + October + November + April + August + imp.trend,data = df)
  return(fit)
}

#############################################################################
# Durbin-Watson Test Function
#
# Purpose: Applies the Durbin-Watson test to a model to assess the presence of autocorrelation in the residuals.
# Input: model, a fitted model object.
# Output: The result of the Durbin-Watson test.
#
# For this study, the months of Sept, Oct, Nov, April and August are significant
#############################################################################

model_dwt<-function(model){dwt (model, max.lag =12, alternative = "two.sided")}



#######################################################################
# Residual Plot Function
#
# Purpose: Generates a scatter plot of OLS model residuals over time.
# Input: df (data frame), model (fitted model object), and color1 (color for the points).
# Output: A plot displaying the residuals of the OLS model.
#############################################################################

graph.ols.residuals<-function(df,model,color1="red"){
  plot (df$time_months[1:74],
        residuals(model)[1:734],
        type='o',
        pch = 20,
        xlab = 'time_months',
        ylab = 'ols residuals',
        col = color1)
  abline(h=0, lty=2)}


###################################################################
# ACF Plot Function
#
# Purpose: Displays autocorrelation and partial autocorrelation plots for the residuals of a fitted model.
# Input: model, a fitted model object.
# Output: Side-by-side ACF and PACF plots for model diagnostics.
##################################################################

graph.ols.acf.plots<-function(model){
  #set plotting to 2 records on one page

  par(mfrow=c(1,2))

  acf(residuals(model))
  acf(residuals(model), type = 'partial')

  #plot one record on one page
  par(mfrow=c(1,1))
}



#############################################################################
# GLS Model Fitting Function
#
```

```
# Purpose: Fits a Generalized Least Squares (GLS) model to the data with options for including squared or cubed time terms and specifying ARMA(p,q)
correlation structures.
# Input: df (data frame with data), timesquared and timecubed (logical flags for including time squared/cubed terms), and p, q (parameters for ARMA
correlation structure).
# Output: A fitted gls model.
#############################################################################

gls.modeller<-function(df,timesquared=FALSE,timecubed=FALSE,p=NULL,q=NULL){


  # for cases where p and q are null
  if(timesquared==F & timecubed==F & is.null(p) & is.null(q)) { a<- gls(admiss_quint_prop ~ time_months +  announcement.trend +  September + October +
November + April + August + imp.trend, data = df,method="ML")}
  if(timesquared==T & timecubed==F & is.null(p) & is.null(q)) { a<- gls(admiss_quint_prop ~ time_months +  announcement.trend +  September + October +
November + April + August + imp.trend, data = df,method="ML")}
  if(timesquared==F & timecubed==T & is.null(p) & is.null(q)) { a <-gls(admiss_quint_prop ~ time_months + announcement.trend +   September + October +
November + April + August + imp.trend, data = df,method="ML")}

  # for cases where p is provided
  if(timesquared==F & timecubed==F & !is.null(p) & is.null(q)) { a<- gls(admiss_quint_prop ~ time_months +  announcement.trend +  September + October +
November + April + August + imp.trend, data = df,correlation= corARMA(p=p, form = ~ time_months),method="ML")}
  if(timesquared==T & timecubed==F & !is.null(p) & is.null(q)) { a<- gls(admiss_quint_prop ~ time_months +  announcement.trend  + September + October +
November + April + August + imp.trend + time_months^2, data = df,correlation= corARMA(p=p, form = ~ time_months),method="ML")}
  if(timesquared==T & timecubed==F & !is.null(p) & is.null(q)) { a<- gls(admiss_quint_prop ~ time_months +  announcement.trend  +  September + October
+ November + April + August + imp.trend + time_months^3 , data = df,correlation= corARMA(p=p, form = ~ time_months),method="ML")}

  #for cases where q is provided
  if(timesquared==F & timecubed==F & is.null(p) & !is.null(q)) { a<- gls(admiss_quint_prop ~ time_months  + announcement.trend + September + October +
November + April + August + imp.trend , data = df,correlation= corARMA(q=q, form = ~ time_months),method="ML")}
  if(timesquared==T & timecubed==F & is.null(p) & !is.null(q)) { a<- gls(admiss_quint_prop ~ time_months  + announcement.trend + September  + October +
November + April + August + imp.trend + time_months^2, data = df,correlation= corARMA(q=q, form = ~ time_months),method="ML")}
  if(timesquared==F & timecubed==T & is.null(p) & !is.null(q)) { a<- gls(admiss_quint_prop ~ time_months  + announcement.trend + September + October +
November + April + August + imp.trend + time_months^3 , data = df,correlation= corARMA(q=q, form = ~ time_months),method="ML")}

  # for cases where both p and q are provided
  if(timesquared==F & timecubed==F & !is.null(p) & !is.null(q)) { a<- gls(admiss_quint_prop ~ time_months  + announcement.trend + September + October +
November + April + August + imp.trend,  data = df,correlation= corARMA(p=p, form = ~ time_months),method="ML")}
  if(timesquared==T & timecubed==F & !is.null(p) & !is.null(q)) { a<- gls(admiss_quint_prop ~ time_months  + announcement.trend + September + October +
November + April + August + imp.trend + time_months^2, data = df,correlation= corARMA(p=p,q=q, form = ~ time_months),method="ML")}
  if(timesquared==F & timecubed==T & !is.null(p) & !is.null(q)) { a<- gls(admiss_quint_prop ~ time_months  + announcement.trend + September + October +
November + April + August + imp.trend + time_months^3 , data = df,correlation= corARMA(p=p,q=q, form = ~ time_months),method="ML")}

  return(a)
}


#############################################################################
# GLS Results Table Function
#
# Purpose: Creates a formatted table summarizing the results of a GLS model.
# Input: df, a fitted gls model, and caption, a string for the table caption.
# Output: A flextable object displaying the model's coefficient estimates, standard errors, t-values, and p-values.
#############################################################################

gls.table.maker<-function(df,caption=""){

  df.results<-round(as.data.frame(summary(df)$tTable),5)
  df.results$variable<-rownames(df.results)
  df.results<-select(df.results,"variable","Value","Std.Error","t-value","p-value")
  flextable(df.results)
  df.results<-flextable(df.results)
  return(df.results)
}


##############################################################################
##############################################################################
# Counterfactual Function
#
# Purpose: These functions (counterfactual.function) computes estimated values (counterfactuals) based on the model's coefficients, reflecting
scenarios without certain interventions.
# Input: Indices for specific observations, the data frame, and a fitted model.
# Output: The counterfactual estimates.
#
# Scenario : CF is no intervention
##############################################################################

counterfactual.function<-function(i,df,model){
  a<- sum(
    (model$coef[1]),                        #add intercept for admiss_quint_prop
    (model$coef[2] * df$time_months[i]),     #add effect of drinks across time
    (model$coef[4] * df$September[i]),       #add effect of September
    (model$coef[5] * df$October[i]),         #add effect of October
    (model$coef[6] * df$November[i]),        #add effect of November
    (model$coef[7] * df$April[i]),           #add effect of April
    (model$coef[8] * df$August[i]))          #add effect of August

  as.numeric(a)
}

##############################################################################
##############################################################################
# Counterfactual Function
#
# Purpose: These functions (counterfactual.function) computes estimated values (counterfactuals) based on the model's coefficients, reflecting
scenarios without certain interventions.
# Input: Indices for specific observations, the data frame, and a fitted model.
# Output: The counterfactual estimates.
#
# Scenario : CF is an announcement, but law does not come in to force
# i.e. there was an announcement, but no 'implementation'
##############################################################################

counterfactual2.function<-function(i,df,model){
  a<- sum(

    (model$coef[1]),                          #add intercept for admiss_quint_prop
    (model$coef[2] * df$time_months[i]),       #add effect of drinks across time
    (model$coef[4] * df$September[i]),         #add effect of September
    (model$coef[5] * df$October[i]),           #add effect of October
    (model$coef[6] * df$November[i]),          #add effect of November
    (model$coef[7] * df$August[i]),            #add effect of August
    (model$coef[3]*df$announcement.trend[i]))  #add change drinks across time after reformulation

    as.numeric(a)
}
```

```
################################################################################
# ITS Model Plot Function
#
# Purpose: Visualizes the fit of an Interrupted Time Series (ITS) model, including actual vs. predicted values and confidence intervals.
# Input: df (data frame), model (fitted model object), visualization parameters, and titles.
# Output: A ggplot object showing the ITS analysis results.
################################################################################

its.model.plot<-function(df,model,ylim.low,ylim.high,points=TRUE, its.title ="", its.ytitle =""){

  a <- ggplot (df,aes(measurement_date,admiss_quint_prop))+
    geom_point(size=0.4,alpha=0.5)+
    geom_vline(xintercept = as.Date("2016-03-15","%Y-%m-%d"),linetype = 2,alpha=0.7, lwd = 1,  color = "black") +
    geom_vline(xintercept = as.Date("2018-04-01", "%Y-%m-%d"),linetype = 6 ,alpha=0.9, lwd = 1, color = "black") +
    geom_ribbon(aes(ymin=ci.min,ymax=ci.max,colour=NULL),fill="grey",alpha=0.6)+

    theme(legend.position = "none") +

    scale_x_date(expand = c(0, 0)) +
    ylim (ylim.low, ylim.high) +
    labs(x = its.title) +
    labs(y = its.ytitle) +
    theme_bw() +
    geom_line(data = df,aes(measurement_date,cf),lwd=1.0,lty=1,color="red") +
    geom_line(aes(measurement_date, predicted),lwd=1.0,color="dark blue")

  return(a)
}


################################################################################
# Predicted Difference Functions
#
# Purpose : Functions  get.predicted.anyweek and get.predicted.anyweek.cf2 calculate the difference between actual and counterfactual
# (or predicted) values at specified time points, useful for assessing the impact of interventions.
################################################################################


get.predicted.anyweek<-function(df,absrel="abs",pos){
  if(absrel=="abs"){
    x <- diff(c(df$cf[pos],df$predicted [pos]))
    x2<- diff(c(df$cf[pos],df$ci.min [pos]))
    x3<- diff(c(df$cf[pos],df$ci.max [pos]))
    x4<- round(100*(x/df$cf[pos]),2)
    x5<- round(100*(x2/df$cf[pos]),2)
    x6<- round(100*(x3/df$cf[pos]),2)

    x<-paste0(round(x,2),", 95% CI ",round(x3,2),",",round(x2,2),") | ", x4,"%, 95% CI ",x5,"%,",x6,"%)  ")

  }

  return(x)

}


get.predicted.anyweek.cf2<-function(df,absrel="abs",pos){
  if(absrel=="abs"){
    x <- diff(c(df$cf2[pos],df$predicted [pos]))
    x2<- diff(c(df$cf2[pos],df$ci.min [pos]))
    x3<- diff(c(df$cf2[pos],df$ci.max [pos]))
    x4<- round(100*(x/df$cf2[pos]),2)
    x5<- round(100*(x2/df$cf2[pos]),2)
    x6<- round(100*(x3/df$cf2[pos]),2)

    x<-paste0(round(x,2),", 95% CI ",round(x3,2),",",round(x2,2),") | ", x4,"%, 95% CI ",x5,"%,",x6,"%)  ")

  }

  return(x)

}
```

MRC Epidemiology Unit, University of Cambridge

Version 1.01 - 2024-05-10

Open Source Licenses

To the best of our knowledge, all code used in this analysis is open source. All derivative code provided here inherits the license terms of the various R packages used in the analyses. Any **novel** code not covered by other licenses is provided on the MIT License*. Please check carefully.

*Copyright (c) <2024> <Nina Rogers (Nina.Rogers@mrc-epid.cam.ac.uk), Chrissy Roberts (Chrissy.Roberts@LSHTM.ac.uk)>

Permission is hereby granted, free of charge, to any person obtaining a copy of this software and associated documentation files (the "Software"), to deal in the Software without restriction, including without limitation the rights to use, copy, modify, merge, publish, distribute, sublicense, and/or sell copies of the Software, and to permit persons to whom the Software is furnished to do so, subject to the following conditions:

The above copyright notice and this permission notice shall be included in all copies or substantial portions of the Software.
