# Supplementary material for: The UK Soft Drinks Industry Levy and childhood hospital admissions for asthma in England
Source: Nat Commun. 2024 Jun 10;15:4934. doi: 10.1038/s41467-024-49120-4 (PMC11164966; doi:10.1038/s41467-024-49120-4)
Supplement: Supplementary file 4 — Description of Additional Supplementary Files [file 41467_2024_49120_MOESM4_ESM.pdf]

Supplementary Software:

This supplement provides copies of all R code and associated outputs that were created in the study. The original analyses used a combination of R Markdown (for analyses) and R scripts (Providing functions and data preparation).
